# Molecular Phylogeny and Evolution of Parabasalia with Improved Taxon Sampling and New Protein Markers of Actin and Elongation Factor-1α

**DOI:** 10.1371/journal.pone.0029938

**Published:** 2012-01-09

**Authors:** Satoko Noda, Cléa Mantini, Dionigia Meloni, Jun-Ichi Inoue, Osamu Kitade, Eric Viscogliosi, Moriya Ohkuma

**Affiliations:** 1 Microbe Division/Japan Collection of Microorganisms, RIKEN BioResource Center, Wako, Saitama, Japan; 2 Interdisciplinary Graduate School of Medicine and Engineering, University of Yamanashi, Kofu, Yamanashi, Japan; 3 Center for Infection and Immunity of Lille, Institut Pasteur de Lille, Lille, France, and Inserm U1019, CNRS UMR 8204, and University Lille – Nord de France, Lille, France; 4 Department of Biomedical Sciences, Division of Experimental and Clinical Microbiology, University of Sassari, Sassari, Italy; 5 Natural History Laboratory, College of Science, Ibaraki University, Mito, Ibaraki, Japan; East Carolina University, United States of America

## Abstract

**Background:**

Inferring the evolutionary history of phylogenetically isolated, deep-branching groups of taxa—in particular determining the root—is often extraordinarily difficult because their close relatives are unavailable as suitable outgroups. One of these taxonomic groups is the phylum Parabasalia, which comprises morphologically diverse species of flagellated protists of ecological, medical, and evolutionary significance. Indeed, previous molecular phylogenetic analyses of members of this phylum have yielded conflicting and possibly erroneous inferences. Furthermore, many species of Parabasalia are symbionts in the gut of termites and cockroaches or parasites and therefore formidably difficult to cultivate, rendering available data insufficient. Increasing the numbers of examined taxa and informative characters (e.g., genes) is likely to produce more reliable inferences.

**Principal Findings:**

Actin and elongation factor-1α genes were identified newly from 22 species of termite-gut symbionts through careful manipulations and seven cultured species, which covered major lineages of Parabasalia. Their protein sequences were concatenated and analyzed with sequences of previously and newly identified glyceraldehyde-3-phosphate dehydrogenase and the small-subunit rRNA gene. This concatenated dataset provided more robust phylogenetic relationships among major groups of Parabasalia and a more plausible new root position than those previously reported.

**Conclusions/Significance:**

We conclude that increasing the number of sampled taxa as well as the addition of new sequences greatly improves the accuracy and robustness of the phylogenetic inference. A morphologically simple cell is likely the ancient form in Parabasalia as opposed to a cell with elaborate flagellar and cytoskeletal structures, which was defined as most basal in previous inferences. Nevertheless, the evolution of Parabasalia is complex owing to several independent multiplication and simplification events in these structures. Therefore, systematics based solely on morphology does not reflect the evolutionary history of parabasalids.

## Introduction

The phylum Parabasalia, or commonly parabasalids, comprises a monophyletic but complex assemblage of diverse species of flagellated protists typified by the presence of a characteristic parabasal apparatus (Golgi complex associated with striated fibers), closed mitosis with an external spindle (cryptopleuromitosis), and anaerobic energy-generating organelles called hydrogenosomes [1], [2]. Based on morphological characters, more than 80 genera and 400 parabasalid species have been described thus far [1], [3]. Most parabasalids inhabit the digestive tract of animal hosts as commensals, parasites, or symbionts. In particular, symbiotic parabasalids found in the gut of termites and wood-eating cockroaches play a central role in the digestion of cellulose [4]. This symbiotic relationship is considered a key element in the evolution of social behavior in the hosts [5] and has ecological significance for the decomposition of plant litter in terrestrial ecosystems [6]. Several parabasalids are also of considerable medical and veterinary importance as pathogens—i.e., *Trichomonas vaginalis* and *Tritrichomonas foetus* [7], [8].

In addition to the functional host interactions of parabasalids, their biodiversity, conspicuous morphology, unique anaerobic biochemistry, and potentially crucial position in various schemes of eukaryotic evolution have long fascinated researchers. Indeed, Parabasalia is included in the supergroup Excavata and in Metamonada with Fornicata (e.g., *Giardia*) and Preaxostyla (e.g., *Monocercomonoides* and *Trimastix*) [9]. The monophyly of Excavata, although supported morphologically [9], [10], is debated still intensely, but multigene analyses interestingly suggest that Excavata stems from a very deep branching event within the history of eukaryotes [11]–[13].

Molecular phylogenetic studies based on small-subunit (SSU) rRNA gene sequences have focused mainly on cultured representatives of parabasalids [14]–[27]. In contrast, molecular studies of the parabasalids found in the gut of termites have been impeded because these organisms live in complex microbial communities and are very difficult to culture [28], [29]. SSU rRNA gene sequences from parabasalid symbionts, however, have been identified subsequently by culture-independent studies [30]–[50] (see also Table 1 for reference in each parabasalid species).

**Table 1:** Parabasalian species used for the gene identification and phylogenetic analyses.

Species	Class^a^	Family^b^	Host animal	SSU rRNA gene reference	GAPDH reference	Method for gene identification for actin and EF-1α
*Macrotrichomonas* sp.	C	L (D)	*Glyptotermes satsumensis*	[46]	[46]	RT-PCR, pooled cells
*Metadevescovina cuspidata*	C	L (D)	*Incisitermes minor*	[46]	[46]	PCR/WGA, pooled cells
*Foaina nana*	C	L (D)	*Cryptotermes domesticus*	[46]	[46]	RT-PCR (actin), PCR/WGA (EF-1α), pooled cells
*Caduceia versatilis*	C	L (D)	*Cryptotermes cavifrons*	[39]	[46]	RT-PCR, pooled cell
*Devescovina* sp.	C	L (D)	*Neotermes koshunensis*	[35]	[55]	RT-PCR, pooled cells
*Gigantomonas herculea*	C	L (D)	*Hodotermes mossambicus*	[46]	[46]	PCR/WGA (EF-1α), two cells^c^
*Stephanonympha* sp. CcSt	C	L (C)	*Cryptotermes cavifrons*	[46]	[46]	PCR/WGA, pooled cells
*Stephanonympha* sp. NkSt	C	L (C)	*Neotermes koshunensis*	[35]	[55]	RT-PCR, pooled cells
*Snyderella tabogae*	C	L (C)	*Cryptotermes cavifrons*	[37]	[46]	RT-PCR (actin), PCR/WGA (EF-1α), pooled cells
*Deltotrichonympha* sp.	C	L (De)	*Mastotermes darwiniensis*	[34]	—d	RT-PCR, pooled cells
*Joenina pulchella*	C	L	*Porotermes adamsoni*	[46]	[46]	RT-PCR, pooled cells
*Joenia annectens*	C	L	*Kalotermes flavicollis*	[46]	[46]	RT-PCR, pooled cells
*Joenoides intermedia*	C	L	*Hodotermes mossambicus*	[46]	[46]	RT-PCR, pooled cells
*Spirotrichonympha leidyi*	S	Hl (Sp)	*Coptotermes formosanus*	[35]	[55]	RT-PCR, pooled cells
*Holomastigotoides mirabile*	S	Hl	*Coptotermes formosanus*	[35]	[55]	RT-PCR, pooled cells
*Staurojoenina assimilis*	Tn	St	*Incisitermes minor*	[38]	This study	RT-PCR, pooled cells
*Trichonympha agilis*	Tn	Tn	*Reticulitermes speratus*	[31]	[54]	RT-PCR (actin), PCR/WGA (EF-1α), pooled cells
*Trichonympha* sp.	Tn	Tn	*Hodotermopsis sjoestedti*	[35]	[47]	RT-PCR (actin), PCR/WGA (EF-1α), single cell
*Hoplonympha* sp.	Tn	Hp	*Hodotermopsis sjoestedti*	[38]	This study	RT-PCR, pooled cells
*Pseudotrichonympha grassii*	Tn	Te (E)	*Coptotermes formosanus*	[35]	[55]	RT-PCR, pooled cells
*Eucomonympha* sp.	Tn	Te (E)	*Hodotermopsis sjoestedti*	[38]	[47]	RT-PCR, single cell
*Teranympha mirabilis*	Tn	Te	*Reticulitermes speratus*	[38]	[47]	RT-PCR, pooled cells
*Histomonas meleagridis*	Tt	Di (M)	*Meleagris gallopavo*	[18]	[56]	PCR/genome DNA
*Dientamoeba fragilis*e	Tt	Di (M)	*Homo sapiens*	[15]	—d	PCR/genome DNA
*Tritrichomonas foetus*	Tt	Tt (Tm)	*Bos primigenus*	[19]	[51]	PCR/genome DNA
*Monocercomonas* sp.	Tt	M	*Natrix sipedon*	[14]	[51]	PCR/genome DNA
*Hypotrichomonas acosta*	H	H (M)	*Drymarchon corais couperi*	[16]	[54]	PCR/genome DNA
*Trichomitus batrachorum*	H	H (Tm)	*Elaphe obsoleta*	[17]	[51]	PCR/genome DNA
*Pentatrichomonas hominis*	Tm	Tm	*Homo sapiens*	[17]	—e	—e
*Tetratrichomonas gallinarum*	Tm	Tm	*Anas platyrhynchos*	[17]	[51]	PCR/genome DNA
*Trichomonas vaginalis*	Tm	Tm	*Homo sapiens*	[14]	[51] e	Reference [74], [75] e

a Abbreviations of the classes are: C, Cristamonadea; Tt, Tritrichomonadea; S, Spirotrichonymphea; H, Hypotrichomonadea; Tm, Trichomonadea; and Tn, Trichonymphea.

b When the family name has changed in the new parabasalian classification [26], the corresponding former name [1] is also indicated in parenthesis. Abbreviations of the families are the following: L, Lophomonadidae; D, Devescovinidae; C, Calonymphidae; De, Deltotrichonymphidae; Sp, Spirotrichonymphidae; Hl, Holomastigotoididae; St, Staurojoeninidae; Tn, Trichonymphidae; Hp, Hoplonymphidae; E, Eucomonymphidae; Te, Teranymphidae; Di, Dientamoebidae; Tt, Tritrichomonadidae; M, Monocercomonadidae; and Tm, Trichomonadidae.

c We failed to identify the EF-1α gene in *G. herculea*.

d The GAPDH genes of *Deltotrichonympha* sp. and *D. fragilis* were unavailable.

e The EST data of *P. hominis* and the genome sequence of *T. vaginalis* [73] in the database were also used for phylogenetic analyses (see Materials and Methods).

Because of a high level of sequence divergence of the SSU rRNA gene across parabasalid lineages, a lack of resolution and possible tree-construction artifacts have occurred, even though a large number of taxa have been investigated [26], [39], [43], [46]. To overcome this drawback, multiple protein-encoding genes such as glyceraldehyde-3-phosphate dehydrogenase (GAPDH), enolase, and tubulins have been examined [51]–[57]. In these phylogenetic reconstructions, however, taxon sampling was limited, and some conflicting results for relationships of major parabasalid groups were obtained depending on the genes. Worse, in the case of enolase, paralogous copies resulting from ancient duplications might confound phylogenetic interpretation [53]–[55], [57]. Among these indicators, GAPDH sequences that contain a greater phylogenetic signal have given well-resolved trees largely congruent with the SSU rRNA gene phylogeny. Consequently, the GAPDH sequences have been used gradually in phylogenetic reconstructions, and the data of many important taxa have accumulated to cover a wide range of parabasalid diversity [46], [47].

Taxonomic classifications of parabasalids have been proposed on the basis of marked morphological differences, particularly in the arrangement of the basal bodies of the flagella and associated cytoskeletal elements [1], [58], [59]. Parabasalids historically have been divided into two classes: Trichomonada and Hypermastigia [1]. Hypermastigia (or hypermastigids) comprises species of typically large-cell forms equipped with numerous flagella, whereas Trichomonada cells are usually smaller and simpler than those of hypermastigids, with up to six flagella. Molecular studies provide critical and sometimes unexpected insights into the evolution of parabasalids and globally conflict with established systematics. For instance, hypermastigids have been considered to have a polyphyletic origin; therefore, many authors have pointed out the need to revise parabasalid systematics on the basis of these molecular data [16], [17], [20], [37], and indeed some systematic revisions have been started [60], [61]. Consequently, Cepicka *et al.* [26] have proposed dividing the parabasalids into six classes: Trichonymphea, Spirotrichonymphea, Cristamonadea, Tritrichomonadea, Hypotrichomonadea, and Trichomonadea. The traditional hypermastigids are assigned to the former three classes, which almost exclusively comprise species occurring in the gut of termites and wood-eating cockroaches. Nevertheless, these revisions do not solve all the problems with the systematics of parabasalids, and great uncertainty remains with respect to the phylogenetic relationships among and within these classes [20], [24], [26], [38], [39], [46], [57].

In phylogenetic analyses based on SSU rRNA gene or concatenated data of protein sequences, Trichonymphea, the most morphologically complex group of parabasalids, frequently occurs as the most basal lineage of Parabasalia [31]–[33], [35], [54], [55]. Support for this rooting often has been poor, however, and the differences among several alternative positions in parabasalid lineages have been insignificant [20], [33], [35], [55]. This uncertainty has been explained by the absence of any close outgroup species to Parabasalia [20]. The parabasalid lineage usually branches out deeply in eukaryote phylogenies, and the branch leading to Parabasalia is very long. Such a long branch may attract the fast-evolving Trichonymphea, causing artificial rooting. Furthermore, the gene encoding GAPDH, like many other glycolytic enzymes, of Parabasalia has been derived from a bacterium *via* lateral gene transfer (LGT) [51], [62]–[64], which disturbs root inference in comparisons to other eukaryotes.

Approaches using the sequence data of multiple genes increase the number of informative characters for phylogenetic inference. In parallel, increasing the number of sampling taxa (or species) is also important. Assembling datasets rich in both genes and taxa is likely to produce more accurate and robust results, although controversy exists about which strategy (increasing the number of genes or taxa) contributes most to the accurate inference [65]–[67]. In general, recent genome sequencing efforts have increased the number of genes for some species, but the genome analyses of either cultured microbial species or yet-uncultivated species are still poor. In terms of taxon sampling, data availability is also restricted, particularly for yet-uncultivated species. The goal of this study is to improve the phylogenetic framework of the relationships among parabasalid groups as well as of the evolution of parabasalid biodiversity by examining two additional genes encoding actin and elongation factor (EF)-1α in diverse parabasalid taxa of both cultivated and yet-uncultivated species. These two proteins are functionally independent from each other and from GAPDH. Their genes are expressed usually highly, and this feature is particularly important for obtaining gene sequences from a small number of manually isolated cells of yet-uncultivated species by reverse transcription (RT)-PCR and designing PCR primers based on EST (expressed sequence tag) data of termite-gut symbionts [68], [69]. Protein sequences of these two molecular markers were examined in representative species that have been investigated already with both SSU rRNA gene and GAPDH. In particular, we examined many members of Cristamonadea and Trichonymphea because both classes contain a large number of genera that were relatively easy to distinguish owing to their conspicuous morphologies. We inferred the phylogenetic relationships among parabasalids and examined the root position of this protistan group based on the concatenation of SSU rRNA gene, actin, EF-1α, and GAPDH sequences.

## Results

### Actin, EF-1α, and GAPDH sequences

In this study, sequences of genes encoding actin and EF-1α were determined from 29 species of parabasalids (Table 1). Among these species, 22 are symbionts in the gut of termites, whereas seven were cultured representatives from other animals. For each gene, several sequences showing more than 98% amino acid identity were obtained from most of these species. These differences can be explained by sequence variation among duplicated gene copies in the genome, at least in the cases of single-cell analyses, and/or by intra-species variation of the gene in the population when multiple cells were used for the analysis. The GAPDH sequences were also obtained from two previously unstudied species, *Hoplonympha* sp. and *Staurojoenina assimilis* because they have been argued to be basal in Trichonymphea [70]–[72]. Two distinct sequences with 89% amino acid identity were obtained from the latter species, which is typical for the GAPDH gene in parabasalids [46], [55]. Maximum protein sequence differences between parabasalian species were 23%, 33%, and 45% for actin, EF-1α, and GAPDH, respectively.

**Figure 1:**
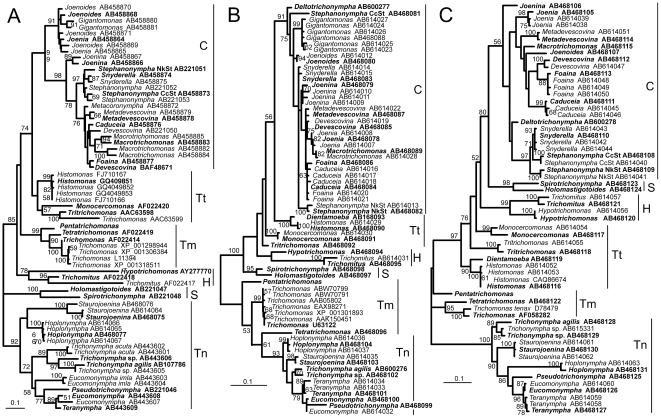
Maximum likelihood phylogenetic analyses of parabasalids based on GAPDH (A), actin (B), and EF-1α (C) sequences. Unambiguously aligned protein sequences of 278 (A), 280 (B) and 274 (C) sites were used for phylogenetic inference. The species names of the parabasalids except for the genus *Trichonympha* are shown in Table 1. The GAPDH sequences of *Trichonympha acuta* and *Eucomonympha imla* and the EF-1α sequence of *Trichomonas tenax* (not shown in Table 1) were also included in the analyses. Although the EF-1α sequences published for *Pentatrichomonas* and *Tritrichomonas* [57] were not included in C because of their shorter sequence length, the analysis with a reducing number of sites (219 sites) demonstrated that they were related very closely to the sequences from the same taxa shown in C. The sequence accession number was indicated for each taxon. The sequences used for the concatenation are in bold. The trees were estimated in RAxML and the numbers near the nodes indicate the bootstrap values. Values below 50% are not shown. Vertical bars to the right of the trees represent the parabasalian classes according to [26]: C, Cristamonadea; S, Spirotrichonymphea; Tn, Trichonymphea; Tt, Tritrichomonadea; H, Hypotrichomonadea; and Tm, Trichomonadea. Scale bars correspond to 0.10 substitutions per site.

Figure 1 shows the phylogenetic trees inferred from the sequences of the three proteins. The sequences of the multiple gene copies identified in this study as well as those found in the genome of *T. vaginalis* [73] showed close phylogenetic relatedness, and thus did not confound the phylogenetic reconstruction—at least the reconstruction needed to infer the relationships among major parabasalian groups, although some inter- or intra-species relationships seemed to be difficult to clarify. GAPDH yielded a relatively good resolution, with many of the branches receiving high statistical support, whereas the actin and EF-1α trees were resolved rather poorly, particularly within Cristamonadea in both cases and within Trichonymphea in the case of actin. Spirotrichonymphea, Trichonymphea, Cristamonadea, and Hypotrichomonadea all were monophyletic, with significant supports for the former two in the three trees. Tritrichomonadea and Trichomonadea were paraphyletic in both the actin and EF-1α trees. A remarkable difference among the three protein-based trees was the position of Spirotrichonymphea. Indeed, it was sister to Trichonymphea in the GAPDH tree and to Cristamonadea in the EF-1α tree (both were supported but only fairly), whereas it was sister to neither Trichonymphea nor Cristamonadea in the actin tree. Depending on the placement of Spirotrichonymphea, relationships among the six parabasalian classes were considerably different for the three proteins, except that Cristamonadea and Tritrichomonadea were grouped together in the GAPDH and actin trees and the group of Trichomonadea and Trichonymphea was separated from the other classes in the actin and EF-1α trees. The results indicated that the analysis of single proteins gave poor resolution and would cause incorrect inferences of relationships because it is unclear which of the individual sequences resolves the species phylogeny most accurately.

### Sequence concatenation and relationships among parabasalian classes

**Figure 2:**
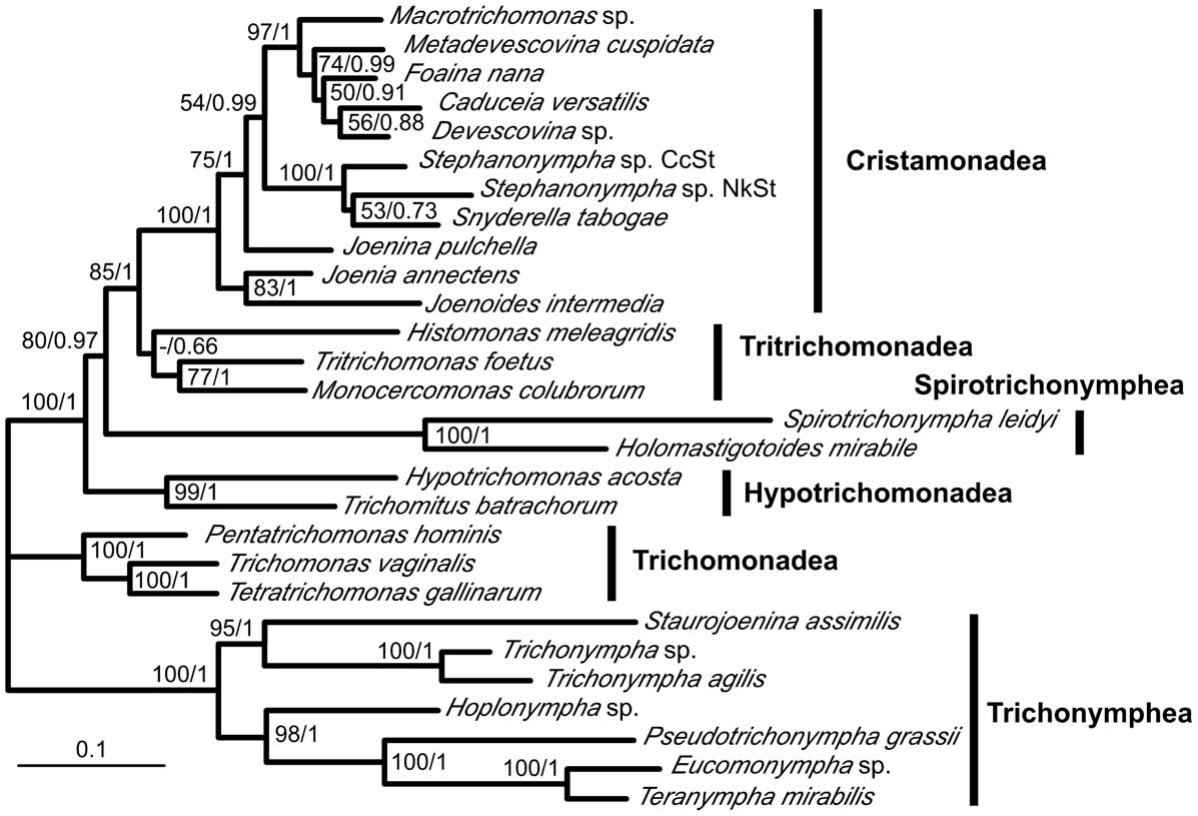
Phylogenetic relationship of parabasalids inferred from the concatenated dataset. The concatenated dataset comprising 278 amino acid sites of GAPDH, 280 amino acid sites of actin, 274 amino acid sites of EF-1α, and 1338 nucleotide sites of SSU rRNA gene sequences was analyzed in 28 parabasalian species. The tree was estimated in RAxML using separate models with the parameters and branch length optimized for each gene partitions individually. The supporting values (bootstrap in RAxML/Bayesian posterior probability) are indicated at the nodes. Values below 50% or 0.5 are indicated with hyphens. When the site-heterogeneous CAT model was used in each partition, the identical tree topology with similar bootstrap values was obtained (data not shown). Vertical bars to the right of the tree represent the parabasalian classes. The scale bar corresponds to 0.10 substitutions per site.

To overcome the poor resolution with single markers, one representative of the multiple gene copies in each of the 28 common parabasalian species was used for sequence concatenation of the three proteins and SSU rRNA gene and subsequent analysis (Figure 2). A significant resolution—particularly in the relationships among the six parabasalian classes (>80% bootstrap values)—was obtained from this concatenated dataset. The monophyly of each parabasalian class was completely supported except in the Tritrichomonadea. In the apical part of the tree, Cristamonadea and Tritrichomonadea formed a well-supported clade, which formed a sister group with Spirotrichonymphea. These three classes were grouped further with Hypotrichomonadea, and the four were separated completely from Trichonymphea and Trichomonadea.

Alternative phylogenetic relationships of parabasalian classes were examined with the concatenated dataset using the Shimodaira-Hasegawa (SH) and approximately unbiased (AU) tests, in which all possible pairings of the classes that did not appear in the tree based on the concatenated dataset were compared (Table 2). The SH test did not reject any pairing comprised of two from four classes, Cristamonadea, Tritrichomonadea, Spirotrichonymphea, and Hypotrichomonadea, although the AU test did not rejected only two pairing ((C+S) and (S+H), see Table 2). The results suggested that some ambiguities remained in the relationships of these four classes.

**Table 2:** Shimodaira-Hasegawa (SH) and approximately unbiased (AU) tests for alternative monophyletic relationships of parabasalian classes.

Monophyletic constraint	*P* value	
	SH	AU
C+S	0.614	0.161
C+H	0.119	<0.001*
Tt+S	0.426	0.002*
Tt+H	0.182	0.015*
S+H	0.866	0.198
C+Tm	<0.001*	<0.001*
C+Tn	<0.001*	<0.001*
Tt+Tm	<0.001*	<0.001*
Tt+Tn	<0.001*	<0.001*
S+Tm	0.011*	<0.001*
S+Tn	0.020*	0.001*
H+Tm	0.016*	<0.001*
H+Tn	0.067	0.006*

Abbreviations of the classes are shown in the footnote of Table 1 or the legend of Figure 1. Asterisks indicate that the tested monophyly was significantly different from the best ML topology at *P*<0.05. Each of the monophyletic groupings of C+Tt and Tm+Tn appeared in the best ML topology.

Congruency of the inferred relationships of the six parabasalian classes was examined using SH tests. The branching order of the six classes obtained by the sequence concatenation was not significantly worse using all the datasets of single proteins or the SSU rRNA gene (*P*>0.05), whereas the relationships inferred from GAPDH and SSU rRNA gene sequences were rejected using the concatenated dataset (*P* = 0.006 and 0.004, respectively), suggesting some phylogenetic noise in these two datasets. Nevertheless, the removal of either the GAPDH or the SSU rRNA gene sequences from the concatenated dataset did not seriously change the overall relationship of the six classes except that Tritrichomonadea became paraphyletic in both cases and Spirotrichonymphea and Hypotrichomonadea formed a sister group with only a weak support in the case of SSU rRNA gene sequence removal (see additional file 1: Figures S1 and S2).

### Root of Parabasalia

**Figure 3:**
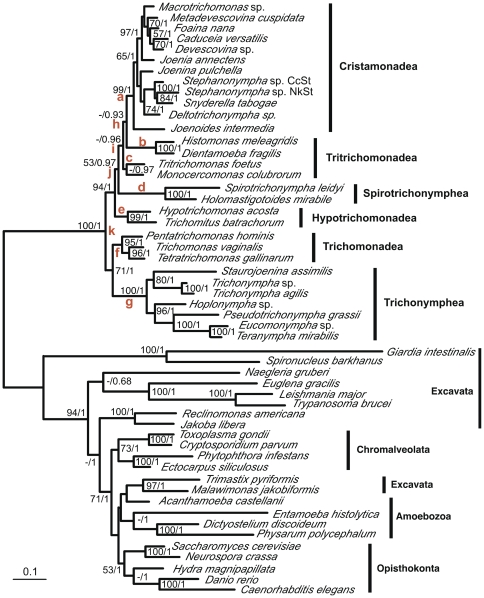
Maximum likelihood tree based on concatenation of actin, EF-1α, and SSU rRNA gene sequences and rooted by diverse eukaryotes. Unambiguously aligned amino acid sites of actin (268) and EF-1α (274), and nucleotide sites of SSU rRNA (1265) gene sequences were concatenated and analyzed in 30 parabasalian species and 23 diverse eukaryotes as outgroups. The tree was estimated with RAxML using the CAT model (CATMIX). The parameters and branch length were optimized for each gene partition individually. The supporting values (bootstrap in RAxML/Bayesian posterior probability) are indicated at the nodes. Values below 50% or 0.5 are indicated with hyphens. Vertical bars to the right of the tree represent the parabasalian classes. The 11 possible root positions are indicated in red letters. The scale bar corresponds to 0.10 substitutions per site.

The root of Parabasalia was investigated through analyses with outgroup taxa. Because the GAPDH gene of Parabasalia likely has been acquired from a bacterium via LGT [51], [62]–[64], a sequence concatenation of EF-1α, actin, and SSU rRNA gene of 30 common parabasalian species was analyzed with representatives of diverse eukaryotic lineages as outgroup taxa (Figure 3). The root of Parabasalia was located at the position dividing the Trichonymphea plus Trichomonadea group from the others (position k in Figure 3). The monophyly of Parabasalia was supported fully. Except for the paraphyly of Tritrichomonadea, the relationships among the parabasalian classes were quite similar to those revealed in the analyses without outgroup taxa, although the supporting values of the branching orders of Cristamonadea, Tritrichomonadea, Spirotrichonymphea, and Hypotrichomonadea were decreased. Notably, each of the groupings of these four classes and the sister-group relationship between Trichomonadea and Trichonymphea had considerable support (94/1 and 74/1, respectively), indicating the significance of the parabasalian root position.

Because some outgroup taxa had very long branches, which might confound the inference of the parabasalian root, these long-branch outgroup taxa were removed from the analyses in a stepwise manner (see Table S1). A series of analyses removing long-branch outgroup taxa from 23 to 13 did not substantially affect the root position, the relationships among the parabasalian classes, or their support values, indicating that these long-branch outgroup taxa did not disturb the inference.

We compared 11 possible root positions (a–k in Figure 3) using the SH and AU tests (Table 3). The root positions at the nodes leading to Trichonymphea (position g), Trichomonadea (f), and Hypotrichomonadea (e), the node dividing the group of these three classes from the others (j), and the clade of *Histomonas* plus *Dientamoeba* (b) were not rejected in the SH test (*P*>0.05), while only the positions g and e were not rejected in the AU test. The results indicated that although the inferred root position k was the most likely, some ambiguity remains.

**Table 3:** Shimodaira-Hasegawa (SH) and approximately unbiased (AU) tests for parabasalian root positions.

Root position	*P* value	
	SH	AU
a	0.012*	0.001*
b	0.088	0.044*
c	0.005*	<0.001*
d	0.019*	0.001*
e	0.367	0.161
f	0.378	0.013*
g	0.491	0.148
h	0.009*	0.002*
i	0.018*	0.001*
j	0.065	<0.001*
k	Best	Best

Root positions are depicted in Figure 3. Asterisks indicate that the root position was significantly different from the best ML topology at *P*<0.05.

**Figure 4:**
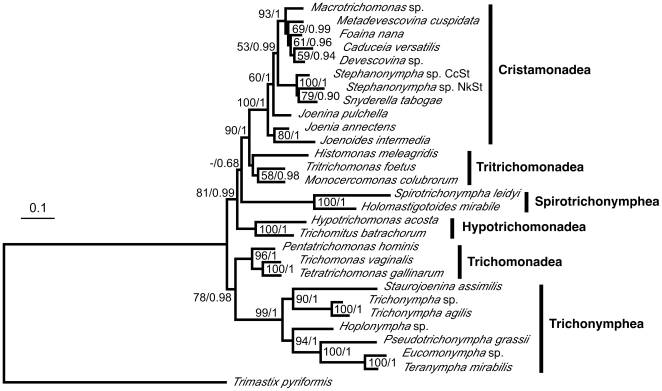
Maximum likelihood tree based on concatenation of GAPDH, actin, EF-1α, and SSU rRNA gene sequences and rooted by *Trimastix*. Unambiguously aligned amino acid sites of GAPDH (257), actin (268), and EF-1α (274), and nucleotide sites of SSU rRNA (1338) gene sequences were concatenated and analyzed in 28 parabasalian species with *Trimastix* as an outgroup. The tree was estimated in RAxML using the CAT model (CATMIX). The parameters and branch length were optimized for each gene partition individually. The supporting values (bootstrap in RAxML/Bayesian posterior probability) are indicated at the nodes. Values below 50% or 0.5 are indicated by hyphens. Vertical bars to the right of the tree represent the parabasalian classes. The scale bar corresponds to 0.10 substitutions per site.

When GAPDH was included in the analysis of the sequence concatenation with a number of eukaryotic taxa as outgroups (data not shown), the identical root position was obtained with considerable support values. Because Preaxostyla such as *Trimastix* is the only known eukaryotic group that shares a common origin of the GAPDH gene of Parabasalia (LGT in their common ancestor or LGTs from closely related bacteria) [62], [63], *Trimastix* was used as an outgroup taxon for parabasalian root analysis of the sequence concatenation including GAPDH (Figure 4). Again, the identical root position was inferred, and significant support values were obtained both at the node uniting Trichonymphea and Trichomonadea and at the node grouping the other four classes. The relationships among the six classes were identical to those shown in Figures 2 and 3.

Addition of α-tubulin and β-tubulin sequences to the concatenated dataset, which reduced the number of the parabasalian taxa available for the analysis to 12, demonstrated that Trichonymphea was the most basal class (see Figure S3), which is consistent with the conclusions of previous studies [26], [55], [56]. To investigate the effects of the number of parabasalian taxa on the inference of their root position, a fixed number of randomly chosen parabasalian taxa were excluded from the concatenated dataset of EF-1α, actin, and SSU rRNA gene sequences and analyzed repeatedly (Table 4). As the number of examined taxa became small, the root position gradually shifted to Trichonymphea, suggesting that the limited numbers of taxa sampling caused the wrong rooting at Trichonymphea.

**Table 4:** Exclusion of parabasalian taxa and the effect on their root.

No. of parabasalian taxa excluded	23 outgroup taxa	13 outgroup taxa
3	10	NT
6	10	NT
12	10	10
16	4	8
18	5	6

Values represent the number of occurrences of root position k (shown in Figure 3) in 10 replicates of the random taxa exclusion analyses in each defined number of excluded taxa. NT, not tested. The 23 outgroup taxa correspond to the concatenate dataset of EF-1α, actin, and SSU rRNA gene sequences using 23 outgroup taxa as shown in Figure 3, whereas the 13 outgroup taxa correspond to those remained after excluding 10 long-branch outgroup taxa (as investigated in Table S1). Note that in the cases of 16 and 18 taxa exclusions, all other replicates demonstrated the root position at the branch leading to Trichonymphea (position g).

## Discussion

### Importance of taxon sampling and new phylogenetic markers

The analyses of many important genera with multi-gene sequences in this study greatly expand our understanding of the evolution of parabasalian biodiversity. Actin and EF-1α sequences, although reported previously in some taxa [57], [74]–[77], are obtained newly in this study in all the examined taxa except for *Trichomonas* and *Pentatrichomonas*. For the first time, the concatenated dataset for 28 or 30 common taxa outlines the robust relationship of most of the major parabasalian groups and a more plausible new root position, thereby largely overcoming the problems encountered in previous molecular phylogenetic studies.

The increased number of parabasalian taxa sampled for the analyses is likely a key parameter in the improvement of Parabasalia rooting. A long-standing debate exists in phylogenetics about whether improved accuracy results from increasing the number of examined taxa (species) or the number of genes (informative characters) [65]–[67], [78]. Studies of empirical data often emphasize the importance of the number of taxa sampled. Particularly, if a small number of taxa that tend to cause long-branch attraction are evaluated with a large number of characters, some slight systematic biases can become magnified and misinterpreted as phylogenetic signals and may cause unfortunately well-resolved, but wrong inferences [78]. In this situation, the addition of taxa in the analysis is an efficient approach. Indeed, multiple changes in an alignment site are detected more easily, and the model parameters for the inference are optimized more precisely when many taxa are analyzed [66], [78].

Previous molecular phylogenetic studies typically have favored rooting at the branch leading to Trichonymphea. Hampl *et al.* [20], however, considered this rooting as an artificially generated wrong inference, even though they did not suggest any robust alternative position. The taxa exclusion analyses described in this study (see Table 4) strongly support the importance of the number of sampled taxa for the inference of the parabasalian root. As the number of examined taxa was reduced, the root gradually shifted to Trichonymphea. The addition of the protein sequences of α- and β-tubulins to the concatenated dataset for the reduced number of taxa (12) unfortunately resulted in rooting at the branch leading Trichonymphea (additional file 1: Figure S3), probably because some systematic biases caused the wrong inference in the limited taxon sampling. Altogether, we conclude that the increased number of sampling taxa improves the accuracy of the rooting of parabasalids, although closely related species to parabasalids as suitable outgroups are still unavailable.

This study provides a significant resolution of the relationship of the major groups of parabasalids. Except for GAPDH, previously examined protein sequences (tubulins and enolase) have been demonstrated to generate only low levels of phylogenetic signals [54], [55], [57], and indeed, concatenated analyses of these protein sequences have yielded conflicting results with poor resolution [26], [55], [56]. Individually, each of these proteins also demonstrated poorly resolved and conflicting relationships of major parabasalian groups (see Figure 1). Therefore, the addition of the actin and EF-1α protein sequences to the sequence concatenation is important for resolution. We conclude that multiple sequences from a single species do not have a strong impact on the phylogenetic analyses based on concatenated sequences. In the enolase case, anciently diverged sequences from a single parabasalid species emerged as two completely distinct positions in the parabasalian phylogeny [53]–[55], [57]. In this study, the sequences from a single species always occurred as close neighbors. The sequences from taxa in a single class formed, in most cases, a monophyletic or at least paraphyletic lineage. The relationships of closely related genera inferred with a certain protein, though poorly resolved, are also observed in many cases in the trees of the other two proteins (see Figure 1). Furthermore, the primers used to amplify actin and EF-1α genes are designed based on EST data of gut symbionts [68], [69] and these primers are matched completely to the EST sequences, but two distinct sequences showing distinct phylogenetic positions have not been obtained at all from a single species. Therefore, this minimized the likelihood that the sequences determined in this study contain anciently diverged paralogs.

Nevertheless, the phylogenetic relationship within some parabasalian classes such as Cristamonadea was resolved still poorly, probably owing to the limited phylogenetic information contained in actin and EF-1α sequences (maximum sequence differences of 17% and 15% between Cristamonadea members, respectively). Moreover, the possibility of several alternative phylogenetic relationships could not be rejected (see Table 2). Further study of other protein markers with sufficient taxon sampling is still needed. Recently, a conserved single-copy gene-encoding largest subunit of RNA polymerase II has been suggested as a useful marker [57]; however, only cultured parabasalids have been investigated so far and studies with yet-uncultivated parabasalids are needed. In the present study, Spirotrichonymphea and Hypotrichomonadea are represented by only two taxa and a further taxon sampling may improve the resolution; however, Hypotrichomonadea comprises only two genera [26] both of which are included in the analyses and Spirotrichonymphea cells, usually small in size, are very hard to distinguish by light microscopy if multiple species occur simultaneously in the gut of termites (and they very often do).

### Phylogenetic relationships among parabasalids and their morphology

These improvements in molecular phylogeny of parabasalids provide us new insights about their evolutionary relationships. A salient point in the present study is the placement of Spirotrichonymphea, which branches out distantly from Trichonymphea and Cristamonadea in the tree based on the sequence concatenation, although the possibility of the sister-group relationship with the latter class cannot be excluded completely. In previous studies, Spirotrichonymphea has been placed ambiguously somewhere among parabasalids. The branch leading to Spirotrichonymphea is very long in phylogenetic trees based on the SSU rRNA gene sequence, suggesting its artificial placement [20], [35], [38]. Some studies have shown its affinity to Cristamonadea [20], [26], [37], and others have located it in a more basal position, near Trichonymphea [35], [55]. In the present study, such conflicting results were obtained in the single-gene phylogenies (see Figure 1).

Historically, Spirotrichonymphea and Trichonymphea have been considered evolutionary closely related to each other because of the similarity of their morphogenesis. Indeed, in both groups, two symmetrical sets of basal bodies, basal fibers, and flagellar bands are separated equally at cell division and then completed in the sister cells [1]. In Spirotrichonymphea, however, flagella are arranged uniquely in left-handed spiral bands originating at the cell apex. Brugerolle [79] has emphasized some common ultrastructural features between Spirotrichonymphea and trichomonads (members in the former family Trichomonadidae), such as the organization of a privileged basal body (#2 in his description) bearing preaxostylar fibers connected to the pelta-axostyle junction. According to our molecular phylogeny based on the concatenated dataset, Spirotrichonymphea and Trichonymphea are neither sister nor sequentially branching lineages. Therefore, we suggest that their common characteristics in morphogenesis have evolved convergently.

In addition to Spirotrichonymphea and Trichonymphea, lophomonads (*Joenia*, *Joenoides*, *Joenina*, and *Deltotrichonympha*; members in the former order Lophomonadida) included within Cristamonadea are parabasalids exhibiting a hypermastigid nature. In our trees, they are related usually distantly to Spirotrichonymphea and Trichonymphea. During cell division, lophomonads retain only four privileged basal bodies, and the flagella are reconstructed in the daughter cells, whereas Spirotrichonymphea and Trichonymphea permanently maintain a multiflagellar state [1]. This feature specific to lophomonads supports their independent emergence from the other two groups. As shown in the present study and previously [46], these lophomonad genera branch out basally in Cristamonadea, although *Deltotrichonympha* forms a distinct lineage from the other lophomonads in SSU rRNA gene sequence analyses [46], [80] and seem to have emerged more recently. In addition, simpler devescovinids and multinucleated calonymphids likely emerged later, and Tritrichomonadea, which is sister to Cristamonadea, contains the most rudimentary *Histomonas* and *Dientamoeba*. Therefore, the apical group of parabasalids comprising Cristamonadea and Tritrichomonadea have undergone dynamic morphological transitions of multiplication and reduction of flagellar and cytoskeletal systems as well as transitions to multinucleated status. Recently, a lophomonad species (*Lophomonas striata*) in a cockroach has been shown to be sister to Trichonymphea, not to nest within Cristamonadea, and thus “lophomnads” are completely polyphyletic [80].

In Trichonymphea, several morphological peculiarities have distinguished the families Hoplonymphidae (*Hoplonympha*) and Staurojoeninidae (*Staurojoenina*) from the other members of this class. Their flagellar areas are restricted to the anterior rostrum and form two and four symmetrical longitudinal rows in Hoplonymphidae and Staurojoeninidae, respectively [1], [70], [81], [82]. In the other Trichonymphea members, flagella in the rostrum form a so-called rostral tube, which is composed of two half-round plates of parabasal fibers. Furthermore, the similarity of Hoplonymphidae and Staurojoeninidae with trichomonads or Spirotrichonymphea members sometimes has been argued [70]–[72]. In our study, relationships within Trichonymphea were resolved fully and Trichonymphea was divided into two robust groups (see Figures 2 and 3). Each of the two groups contains either Hoplonymphidae or Staurojoeninidae as the basal lineage. If flagellar organization in the limited number of longitudinal rows is primitive, the rostral tube and flagella in the post-rostral area likely developed convergently in these two groups.

Our phylogenetic inference mostly corroborates the new classification proposed by Cepicka *et al.* [26]. Former classifications that elevate a group corresponding to Cristamonadea to the ordinal level [60], [61] have resulted in the paraphyly or polyphyly of members outside Cristamonadea, Spirotrichonymphea, and Trichonymphea, as previous studies have stated repeatedly [20], [26], [39], [46]. These members now are divided into the three classes—Tritrichomonadea, Hypotrichomonadea, and Trichomonadea—which comprise trichomonads and monocercomonads (members in the former families Trichomonadidae and Monocercomonadidae, respectively). These three classes clearly form distinct lineages in the trees reported in this study. Therefore, this reclassification indeed marks significant progress for parabasalian systematics. Nevertheless, some uncertainties remain. In our analyses, Tritrichomonadea was either monophyletic or paraphyletic at the base of Cristamonadea, but neither relationship was supported significantly. Because formal taxonomic units should be monophyletic, the revision of the taxonomic status of Tritrichomonadea (and also Cristamonadea) may be necessary as discussed previously [26]; for instance, these two classes can be united when data for more robust phylogenies become available. Moreover, several newly proposed classes have not received monophyletic confidence definitively owing to the lack of robust molecular phylogeny, and molecular phylogenetics often denies morphology-based classifications from family to genus or species levels [26], [44], [46]. A large number of species examined with the SSU rRNA gene sequences, which provide only a low level of phylogenetic resolution, have not yet been analyzed using protein sequences. These species are, for example, *Honigbergiella*, *Ditrichomonas*, *Lacusteria*, and *Hexamastix* (Trichomonadea) or *Simplicimonas* (Tritrichomonadea).

### Evolutionary implications

**Figure 5:**
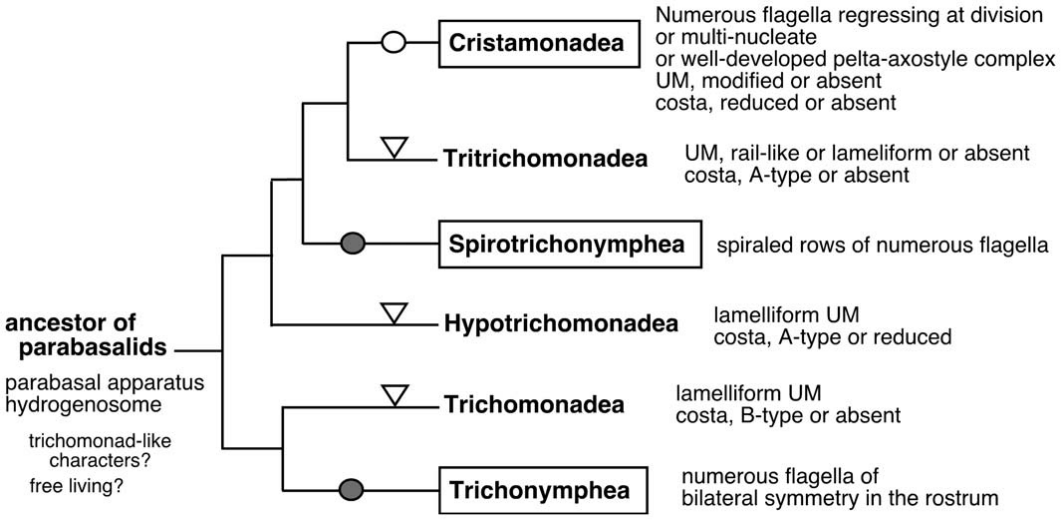
Proposed evolutionary relationships of parabasalids. The tree shows the relationships of the six parabasalian classes. Flagellar multiplication in a single mastigont system has occurred independently in the boxed classes. The multiplications have occurred ancestrally in two classes (marked with filled circles) and probably twice within the other class (open circle). Triangles indicate the occurrence of cytoskeletal simplification in undulating membrane (UM) and costa. See the text for details.

The new root position that we have uncovered suggests that the ancient, most primitive parabasalid has a trichomonad-like character (Figure 5), although other possibilities cannot be excluded. Supporting this conclusion, all the possible alternative basal lineages of parabasalids (root positions b, e, f, and j in Figure 3; see also Table 3) are close to or representative of the classes including trichomonads except for the Trichonymphea lineage, which likely results from an incorrect inference as discussed above. In particular, trichomonads in Tritrichomonadea, Hypotrichomonadea, and Trichomonadea are specified by the presence of a costa and undulating membrane. Their recurrent flagellum is associated with the cell body, forming an undulating membrane underlain by a striated fiber, the costa. The structures of the costa and undulating membrane exhibit variations among these three classes. The costa has a similar banding pattern in members of Tritrichomonadea and Hypotrichomonadea (A-type striation according to Honigberg *et al.* [72]), whereas the costa of Trichomonadea shows a different banding pattern (B-type striation). The undulating membrane is rail-like (in *Tritrichomonas*) or lamelliform (in *Simplicimonas*) in Tritrichomonadea and lamelliform in both Hypotrichomonadea and Trichomonadea. The primitive parabasalid likely possessed the lamelliform undulating membrane, and the rail-like undulating membrane evolved later. The homologous protein components of both types of costa suggest their common origin [83]. The differentiation of the two types of costa probably occurred very early in parabasalid evolution; however, the A-type striation pattern may be primitive because it also occurs in the parabasal fibers of most parabasalids. The common ancestor of parabasalids very likely possessed a parabasal apparatus and hydrogenosomes because they are common characters of parabasalids (Figure 5).

Based solely on comparisons of morphological characters, the most ancient lineage of parabasalids historically has been believed to be monocercomonads owing to their cytoskeletal simplicity, and to the assumption that complex structure and morphology developed later during parabasalian evolution [58], [59]. Earlier molecular phylogenetic studies challenged this simple-to-complex evolutionary scheme, and have suggested almost the reverse scheme owing to the basal placement of the most elaborate Trichonymphea. The new root position described in this study indicates that the evolution of parabasalids is principally simple-to-complex, but the complexity has emerged independently in multiple lineages in different modes of flagellar system multiplication.

Possible occasional reversions to simpler forms make the matter more complicated. Monocercomonads, the most rudimentary parabasalids, are polyphyletic, as clearly shown in the present study as well as by previous work [16], [18], [24]–[26]. Indeed, some monocercomonads such as *Monocercomonas* and *Histomonas* are related closely to *Tritrichomonas*. The monocercomonad *Hypotrichomonas* forms a clade with *Trichomitus*. Other monocercomonads such as *Ditrichomonas* and *Honigbergiella* are likely related to Trichomonadea according to analyses based on SSU rRNA gene sequences [16], [17], [24]. Therefore, secondary reduction of cellular complexity seems to have occurred in each of the three classes—Tritrichomonadea, Hypotrichomonadea, and Trichomonadea (see Figure 5).

A growing number of free-living parabasalids have been investigated recently based on the SSU rRNA gene sequence in *Honigbergiella*, *Lacusteria*, and *Pseudotrichomonas* in addition to *Ditrichomonas* and *Monotrichomonas* [26], [27], although phylogenetic positions of these free-living species was not examined in this study. The free-living species seemingly are dispersed in SSU rRNA gene-based phylogenetic trees but many form a paraphyletic assemblage near the origin of Trichomonadea [27]. Considering the new root position of Parabasalia inferred by this study, it is possible that free-living species represent the most basal lineages of Parabasalia (Figure 5). This possibility needs to be investigated further, because it is of ecological and evolutionary significance for the origin of parabasalids as well as parasitic trichomonads as discussed previously [27].

The hypermastigid nature of flagellar multiplication in a single mastigont has evolved independently at least three times, and these multiplications led to the Trichonymphea, Spirotrichonymphea, and lophomonads in Cristamonadea (see Figure 5). Because these three classes are found only in the gut of termites and cockroaches, each ancestor of these three classes (presumably a trichomonad-like species) established a symbiotic relationship with a host ancestor and evolved and diversified in the gut of their hosts as previously described for Trichonymphea members [47]. Adaptation to the gut environment significantly affects their morphological evolution. Indeed, the development of their flagella and associated cytoskeletal system is advantageous for the improvement of fitness in this niche, because it allows vigorous movement that prevents them from flowing out of the gut and facilitates their access to food in the gut. Their habitats also likely affect cell size, which is associated with cytoskeletal development. The cell of the gut symbionts is large enough to incorporate masticated wood particles by phagocytosis, whereas parasitic and free-living species absorb smaller molecules or tiny bacterial cells as food. Meanwhile, members of the genera *Pseudotrypanosoma* and *Trichomitopsis* in Trichomonadea are also found among the gut symbionts of termites, and their large cell sizes seem to represent a consequence of their adaptation to the gut environment, although they apparently do not develop a complex flagellar system [33], [36].

### Conclusions

This study provides a revised, taxonomically broad phylogenetic framework for Parabasalia. We consider both the increasing number of taxa sampled and the use of new protein markers as particularly important factors in the accuracy and robustness of our inferences. Culture-independent analyses of the termite-gut symbionts are critical for collecting data for the large number of examined taxa, and such techniques are powerful for the investigation of additional data and taxa. The evolution of Parabasalia is complex in terms of morphology owing to a number of independent multiplications and simplifications of flagella and associated cytoskeletal structures. Morphology-based systematics sometimes has hampered the understanding of the true nature of parabasalian evolution. Likely, their ecology greatly affects evolution through adaptation to the niches and co-diversification with their hosts.

## Materials and Methods

### Cultivation and DNA extraction of trichomonads

Culturable strains used were as follows: *Tetratrichomonas gallinarum* strain A6 (cf. [17], [84]); *T. foetus* strain KV1 (ATCC 30924); *Monocercomonas* sp. strain NS-1PRR (ATCC 50210); *Trichomitus batrachorum* strain G11 (ATCC 30066); *Hypotrichomonas acosta* strain L3 (ATCC 30069). The origins of their isolation are shown in Table 1. All strains were grown axenically at 37°C or 27°C in trypticase-yeast extract-maltose (TYM) medium [85] without agar supplemented with 10% (v/v) heat-inactivated horse serum (Gibco-BRL), 100 U/mL of penicillin G, and 50 µg/mL of streptomycin sulfate. Genomic DNA was isolated as described [86]. DNAs of *Dientamoeba fragilis* strain Bi/PA (ATCC 30948) and *Histomonas meleagridis* strain HmZL [18] were provided by C. G. Clark (London School of Hygiene and Tropical Medicine, London, UK) and F. Delbac (LMGE, CNRS UMR 6023, Aubière, France), respectively.

### Manipulation of termite symbionts

Table 1 lists the gut symbionts investigated in this study and their host termites. All taxa were found stably in the hindgut flora of the respective termites and were easily recognizable on the basis of their morphological characters [1], [3]. According to a comprehensive list of flagellate species in the gut of termites [3], cristamonads inhabiting each termite species are as follows: *P. adamsoni* and *I. minor* harbors only *J. pulchella* and *M. cuspidata*, respectively; *H. mossambicus* harbors only *J. intermedia* and *G. herculea*; *K. flavicollis* harbors two *Foaina* spp. in addition to *J. annectens*; *C. cavifrons* harbors species of *Foaina* in addition to the three cristamonads examined in this study; and *C. domesticus* harbors species of *Devescovina* and *Stephanonympha* in addition to *F. nana*. As cristamonad symbionts, *N. koshunensis* harbors species of *Foaina* as well as *Devescovina* and *Stephanonympha* [37], and *G. satsumensis* harbors species of *Foaina* and *Devescovina* in addition to *Macrotrichomonas* sp. [46]. The SSU rRNA and GAPDH genes of the cristamonad species mentioned above (except *Stephanonympha* sp.) were analyzed simultaneously using the same cell preparations [46]. *M. darwiniensis* harbors species of *Koruga*, *Mixotricha*, and *Metadevescovina* in addition to *Deltotrichonympha* [3], and the SSU rRNA gene sequence of *Deltotrichonympha* sp. obtained from our preparation was almost identical to those of *Deltotrichonympha* spp. (AJ583378, AJ583378, and AB326380). *C. formosanus* harbors only three parabasalian species [3], all of which were examined in this study. As species in Trichonymphea that show conspicuous morphology and thus are easily recognizable, *I. minor* harbors only *S. assimilis* [3], *R. speratus* harbors only *T. agilis* and *T. mirabilis* [3], *H. sjoestedti* harbors only species of *Trichonympha*, *Eucomonympha*, and *Hoplonympha* [87]. All of these genera were examined in this study.

*M. darwiniensis*, *P. adamsoni*, *K. flavicollis*, and *H. mossambicus* were collected in Australia, Australia, France, and Kenya, respectively, and generously provided by C. Bordereau (Université de Bourgogne, France). *C. cavifrons* collected in the United States was provided by M. F. Dolan (University of Massachusetts, USA). *I. minor* collected in Japan was provided by W. Ohmura (Forestry and Forest Products Research Institute, Japan). *G. satsumensis*, *C. domesticus*, *C. formosanus*, *R. speratus*, and *H. sjoestedti* were collected in Japan.

The cells of parabasalian symbionts in the hindgut suspension of each termite were isolated manually and washed extensively under a microscope equipped with a micromanipulator (Cell Tram, Eppendorf) as described elsewhere [88], [89]. A single cell or a pool of 10–30 cells showing typical morphology were isolated and used as templates for RT-PCR (see Table 1). In some cases, the isolated cells were subjected to isothermal whole-genome amplification (WGA) as previously described [90], [91], and the amplified genome DNA was used as a template for PCR (see Table 1).

### Cloning and sequencing of actin, EF-1α, and GAPDH genes

The protein-coding genes were amplified using RT-PCR from the isolated cells of termite-gut symbionts using protein-specific primers for the amino-terminal conserved region and the oligo-dT primer as previously described [55]. The design of the actin and EF-1α primers was based on the comparisons of the EST data of the gut symbionts of termites [68], [69] and sequences of various eukaryotes because we found sequence mismatches between previously used primers and the EST data. The following protein-specific primers were used: actin-F1, 5′-TGGGANGANATRGARAARATYTGG3′ and EF1F1, 5′-AARGCDGARCGNGARCGDGG-3′. The protein-specific primers for the carboxy-terminal conserved region used for PCR were actin-R1, 5′-GAAGCAYTTNCKRTGNACDAT-3′ and EF1R1, 5′-GRAAYTTRCANGCDATRTG-3′. If sufficient amplification product was not obtained during the first PCR, a second amplification was performed using the primer actin-F2, 5′-ATRGARAARATYTGGCAYCA-3′ and the oligo-dT primer for the actin gene, and the primer EF1F2, 5′-CGDGGDATYACNATYGAYAT-3′ and the EF1R1 or oligo-dT primer for the EF-1α gene. The GAPDH gene was amplified with RT-PCR using previously designed primers [55]. The amplification products were separated using agarose gel electrophoresis, purified, and cloned into pCR2.1-TOPO (Invitrogen). Clones containing inserts of the expected size were picked and partially sequenced, and the complete DNA sequence of each representative clone was obtained via primer walking. The sequences obtained in this study have been deposited in the DNA Databank of Japan and the accession numbers are shown in Figure 1.

### Phylogenetic analyses

The protein sequences identified in this study and publicly available were used for the analyses. The genome sequence of *T. vaginalis* G3 [73] was searched for homologous sequences with the three proteins. The EST sequences of *Pentatrichomonas hominis* in the public database (FL516063–FL518016) were also searched, and the identified ESTs were assembled to produce *in silico*-translated amino acid sequences. All the non-identical amino acid sequences showing significant identities to sequences of other parabasalids (such as >50% identity) and covering most of the protein region were included in the analyses. In the case of *P. hominis*, only a single unique sequence was found in each protein. The recently reported GAPDH sequences of *H. meleagridis* [56] were also used.

These protein sequences were aligned using ClustalW2 [92] and refined manually. Only unambiguously aligned positions were used for phylogenetic inference. Because any outgroup sequences may cause long-branch attraction owing to their distant relationships to parabasalids, the phylogenetic tree of a single protein was inferred without outgroups. The appropriate model of sequence evolution was selected using the program ProtTest 2.4 [93]. For the tree of each single protein dataset, maximum likelihood (ML) analysis was carried out with RAxML 7.2.6 [94] using the PROTGAMMAWAG model. Four gamma-distibuted substition rate categories were used in this and the following analyses.

The alignments of the protein sequences and previously aligned sequences of the SSU rRNA gene [46] were concatenated manually. When the analyses included GAPDH, the taxa *Deltotrichonympha* sp. and *D. fragilis* were excluded because of the lack of their GAPDH sequences. The outgroup taxa and their sequences used for the analyses are shown in Table S2. The sequence alignments used for the analyses shown in Figures 2 and 3 are available as supplementary data (Datasets S1 and S2).

For the analyses of only parabasalids, the ML tree was estimated in RAxML using mixed models (GTRGAMMA for the SSU rRNA gene and PROTGAMMAWAG for each protein sequence). Parameters and branch length were optimized for each of the partitions individually and bootstrap values were obtained from 1000 replicates. The ML estimation and the bootstrap were also conducted with the site-heterogeneous CAT model in RAxML. Bayesian analysis was performed in MrBayes 3.1.2 [95] using separate models (GTR+I+Γ for the SSU rRNA gene and WAG+I+Γ for each protein sequence), and parameters and branch lengths were optimized for each partition individually. The starting tree was random, and four simultaneous Markov chains in duplicate were run for 10,000,000 generations. Log likelihoods stabilized well before 2,000,000 generations, and the remaining generations were used to measure Bayesian posterior probabilities. The homogeneity of sequence composition in each protein or gene was evaluated in the χ^2^ test implemented in TREE-PUZZLE 5.2 [96].

For the analyses with outgroup taxa, the ML tree was estimated as described above but using the CAT model (GTRMIX and PROTMIXWAG in RAxML) instead of the site-homogeneous model, because the site-heterogeneous CAT model appears to be more robust than site-homogeneous models against artifacts by long-branch attraction [97]. Bootstrap analyses of 1000 replicates were conducted with the CAT model in RAxML. Bayesian analysis was performed in MrBayes as described above.

Differences in alternative tree topologies were compared with the SH test implemented in CONSEL [98] using the site-wise log-likelihood outputs obtained with RAxML. Using only the data from parabasalids, an alternative tree topology was obtained under the constraint of a given phylogenetic hypothesis by the RAxML analysis with the same substitution model described above. For the root of parabasalids, the outgroup was grafted onto 11 possible root positions of parabasalids and tree topology was obtained under each constraint, and these root positions were evaluated using the SH test. To measure the effect of the number of analyzed taxa on the parabasalian root position, randomly chosen parabasalian taxa were excluded and analyzed repeatedly with different sets of fixed numbers of excluded taxa.

## Supporting Information

Figure S1 **Exclusion of GAPDH from the sequence concatenation of parabasalids.** The GAPDH sequence was excluded from the dataset used for the inference shown in Figure 2, and the sequences of *Deltotrichonympha* sp. and *Dientamoeba* were added to the analysis. The tree was inferred using RAxML with the parameters and branch length optimized for each partition. The bootstrap values above 50% are indicated at the nodes. Note that the relationship of the six parabasalian classes and the root position did not change after the exclusion except for the paraphyly of Tritrichomonadea.
(PDF)

Figure S2 **Exclusion of SSU rRNA gene from the sequence concatenation of parabasalids.** The SSU rRNA gene sequence was excluded from the dataset used for the inference shown in Figure 2, and the tree was inferred using RAxML with the parameters and branch length optimized for each partition. The bootstrap values above 50% are indicated at the nodes. Note that the relationship of the six parabasalian classes and the root position did not change after the exclusion except for the paraphyly of Tritrichomonadea and the sister-group relationship of Spirotrichonymphea and Hypotrichomonadea. The removal of the SSU rRNA gene sequence was particularly important because the base composition of this gene was heterogeneous in the two Spirotrichonymphea members and *Histomonas*. All taxa were homogeneous in amino acid composition in the three protein sequences.
(PDF)

Figure S3 **Maximum likelihood tree of the sequence concatenation of actin, EF-1α, α-tubulin, β-tubulin, and SSU rRNA gene in 13 parabasalian taxa and outgroup eukaryotes.** Unambiguously aligned sites of α-tubulin (351) and β-tubulin (145) were concatenated with the dataset used for the analysis shown in Figure 3. The sequence data for both tubulins used for the concatenation are the same as described previously [55]. The tree was inferred using RAxML with the parameters and branch length optimized for each partition. The bootstrap values above 50% are indicated at the nodes. Because the sequences of α- and β-tubulin available for *Pentatrichomonas* and *Entamoeba* are short, we excluded them from the analysis. Trichonymphea was the most basal parabasalian lineage in this analysis; however, this rooting was likely a wrong inference caused by the limited taxon sampling (see the main text). Spirotrichonymphea instead of Tritrichomonadea was sister to Cristamonadea, but this change was not supported at all. The taxa exclusion analyses (see Table 4) indicated that when the number of parabasalian taxa was reduced, the frequency of the pairing of Cristamonadea and Spirotrichonymphea increased up to four times in 10 repeated analyses, suggesting an artificial pairing owing to the limited number of examined taxa.
(PDF)

Table S1 **Removal of long-branch outgroup taxa and the effect on bootstrap values for major nodes within Parabasalia.**
(DOC)

Table S2 **Species used for the outgroup and their sequence accession numbers used for the concatenation.**
(DOC)

Dataset S1 **NEXUS-format alignment of sequence concatenation of GAPDH, actin, EF-1α, and SSU rRNA gene used for the analyses shown in** **Figure 2** **.**
(TXT)

Dataset S2 **NEXUS-format alignment of sequence concatenation of actin, EF-1α, and SSU rRNA gene used for the analyses shown in** **Figure 3** **.**
(TXT)

## References

[1] Brugerolle G, Lee JJ, Lee JJ, Leedale GF, Patterson DJ, Bradbury PC (2001). Phylum Parabasalia.. An illustrated guide to the protozoa. 2nd edition.

[2] Honigberg BM, Brugerolle G, Honigberg BM (1990). Structure.. Trichomonads parasitic in humans.

[3] Yamin MA (1979). Flagellates of the orders Trichomonadida Kirby, Oxymonadida Grassé, and Hypermastigida Grassi & Foà reported from lower termites (Isoptera families Mastotermitidae, Kalotermitidae, Hodotermitidae, Termopsidae, Rhinotermitidae, and Serritermitidae) and from the wood-feeding roach *Cryptocercus* (Dictyoptera: Cryptocercidae).. Sociobiology.

[4] Brune A, Ohkuma M, Bignell DE, Roisin Y, Lo N (2011). Role of the termite gut microbiota in symbiotic digestion.. Biology of termites: a modern synthesis.

[5] Nalepa CA, Bignell DE, Bandi C (2001). Detritivory, coprophagy, and the evolution of digestive mutualisms in Dictyoptera.. Insectes Sociaux.

[6] Ohkuma M (2003). Termite symbiotic systems: efficient bio-recycling of lignocellulose.. Appl Microbiol Biotechnol.

[7] Honigberg BM, Kreier JP (1978). Trichomonads of veterinary importance.. Parasitic protozoa. Volume 2.

[8] Honigberg BM, Kreier JP (1978). Trichomonads of importance in human medicine.. Parasitic protozoa. Volume 2.

[9] Simpson AGB (2003). Cytoskeletal organization, phylogenetic affinities and systematics in the contentious taxon Excavata (Eukaryota).. Int J Syst Evol Microbiol.

[10] Cavalier-Smith T (2003). The excavate protozoan phyla Metamonada Grasse emend. (Anaeromonadea, Parabasalia, Carpediemonas, Eopharyngia) and Loukozoa emend. (Jakobea, Malawimonas): their evolutionary affinities and new higher taxa.. Int J Syst Evol Microbiol.

[11] Hampl V, Horner DS, Dyal P, Kulda J, Flegr J (2005). Inference of the phylogenetic position of oxymonads based on nine genes: support for Metamonada and Excavata.. Mol Biol Evol.

[12] Hampl V, Hug L, Leigh JW, Dacks JB, Lang BF (2009). Phylogenomic analyses support the monophyly of Excavata and resolve relationships among eukaryotic “supergroups”.. Proc Natl Acad Sci U S A.

[13] Simpson AG, Inagaki Y, Roger AJ (2006). Comprehensive multigene phylogenies of excavate protists reveal the evolutionary positions of “primitive” eukaryotes.. Mol Biol Evol.

[14] Gunderson J, Hinkle G, Leipe D, Morrison HG, Stickel SK (1995). Phylogeny of trichomonads inferred from small-subunit rRNA sequences.. J Eukaryot Microbiol.

[15] Silberman JD, Clark CG, Sogin ML (1996). *Dientamoeba fragilis* shares a recent common evolutionary history with the trichomonads.. Mol Biochem Parasitol.

[16] Edgcomb V, Viscogliosi E, Simpson AGB, Delgado-Viscogliosi P, Roger AJ (1998). New insights into the phylogeny of trichomonads inferred from small subunit rRNA sequences.. Protist.

[17] Delgado-Viscogliosi P, Viscogliosi E, Gerbod D, Kulda J, Sogin ML (2000). Molecular phylogeny of parabasalids based on small subunit rRNA sequences, with emphasis on the Trichomonadinae subfamily.. J Eukaryot Microbiol.

[18] Gerbod D, Edgcomb VP, Noël C, Zenner L, Wintjens R (2001). Phylogenetic position of the trichomonad parasite of turkeys, *Histomonas meleagridis* (Smith) Tyzzer, inferred from small subunit rRNA sequence.. J Eukaryot Microbiol.

[19] Tachezy J, Tachezy R, Hampl V, Sedinova M, Vanacova S (2002). Cattle pathogen *Tritrichomonas foetus* (Riedmuller, 1928) and pig commensal *Tritrichomonas suis* (Gruby & Delafond, 1843) belong to the same species.. J Eukaryot Microbiol.

[20] Hampl V, Cepicka I, Flegr J, Tachezy J, Kulda J (2004). Critical analysis of the topology and rooting of the parabasalian 16S rRNA tree.. Mol Phylogenet Evol.

[21] Hampl V, Vrlik M, Cepicka I, Pecka Z, Kulda J (2006). Affiliation of *Cochlosoma* to trichomonads confirmed by phylogenetic analysis of the small-subunit rRNA gene and a new family concept of the order Trichomonadida.. Int J Syst Evol Microbiol.

[22] Cepicka I, Hampl V, Kulda J, Flegr J (2006). New evolutionary lineages, unexpected diversity, and host specificity in the parabasalid genus *Tetratrichomonas*.. Mol Phylogenet Evol.

[23] Hess M, Kolbe T, Grabensteiner E, Prosl H (2006). Clonal cultures of *Histomonas meleagridis*, *Tetratrichomonas gallinarum* and a *Blastocystis* sp. established through micromanipulation.. Parasitology.

[24] Hampl V, Cepicka I, Flegr J, Tachezy J, Kulda J (2007). Morphological and molecular diversity of the monocercomonadid genera *Monocercomonas*, *Hexamastix*, and *Honigbergiella* gen. nov.. Protist.

[25] Mantini C, Dalia-Cornette J, Noda S, Van Der Heijden HM, Capron M (2009). Molecular identification and phylogenetic relationships of trichomonad isolates of galliform birds inferred from nuclear small subunit rRNA gene sequences.. Parasitol Res.

[26] Cepicka I, Hampl V, Kulda J (2010). Critical taxonomic revision of Parabasalids with description of one new genus and three new species.. Protist.

[27] Yubuki N, Céza V, Cepicka I, Yabuki A, Inagaki Y (2010). Cryptic diversity of free-living parabasalids, *Pseudotrichomonas keilini* and *Lacusteria cypriaca* n. g., n. sp., as inferred from small subunit rDNA sequences.. J Eukaryot Microbiol.

[28] Ohkuma M (2008). Symbioses of flagellates and prokaryotes in the gut of lower termites.. Trends Microbiol.

[29] Ohkuma M, Brune A, Bignell DE, Roisin Y, Lo N (2011). Diversity, structure, and evolution of the termite gut microbial community.. Biology of termites: a modern synthesis.

[30] Berchtold M, König H (1995). Phylogenetic position of the two uncultivated trichomonads *Pentatrichomonoides scroa* Kirby and *Metadevescovina extranea* Kirby from the hindgut of the termite *Mastotermes darwiniensis* Froggatt.. Syst Appl Microbiol.

[31] Ohkuma M, Ohtoko K, Grunau C, Moriya S, Kudo T (1998). Phylogenetic identification of the symbiotic hypermastigote *Trichonympha agilis* in the hindgut of the termite *Reticulitermes speratus* based on small-subunit rRNA sequence.. J Eukaryot Microbiol.

[32] Dacks JB, Redfield RJ (1998). Phylogenetic placement of *Trichonympha*.. J Eukaryot Microbiol.

[33] Keeling PJ, Poulsen N, McFadden G (1998). Phylogenetic diversity of parabasalian symbionts from termites, including the phylogenetic position of *Pseudotrypanosoma* and *Trichonympha*.. J Eukaryot Microbiol.

[34] Fröhlich J, König H (1999). Rapid isolation of single microbial cells from mixed natural and laboratory populations with the aid of a micromanipulator.. Syst Appl Microbiol.

[35] Ohkuma M, Ohtoko K, Iida T, Tokura M, Moriya S (2000). Phylogenetic identification of hypermastigotes, *Pseudotrichonympha*, *Spirotrichonympha*, *Holomastigotoides*, and parabasalian symbionts in the hindgut of termites.. J Eukaryot Microbiol.

[36] Keeling PJ (2002). Molecular phylogenetic position of *Trichomitopsis termopsidis* (Parabasalia) and evidence for the Trichomitopsiinae.. Eur J Protistol.

[37] Gerbod D, Noël C, Dolan MF, Edgcomb VP, Kitade O (2002). Molecular phylogeny of parabasalids inferred from small subunit rRNA sequences, with emphasis on the Devescovinidae and Calonymphidae (Trichomonadea).. Mol Phylogenet Evol.

[38] Ohkuma M, Iida T, Ohtoko K, Yuzawa H, Noda S (2005). Molecular phylogeny of parabasalids inferred from small subunit rRNA sequences, with emphasis on the Hypermastigea.. Mol Phylogenet Evol.

[39] Noël C, Noda S, Mantini C, Dolan MF, Moriya S (2007). Molecular phylogenetic position of the genera *Stephanonympha* and *Caduceia* (Parabasalia) inferred from nuclear small subunit rRNA gene sequences.. J Eukaryot Microbiol.

[40] Carpenter KJ, Keeling PJ (2007). Morphology and phylogenetic position of *Eucomonympha imla* (Parabasalia: Hypermastigida).. J Eukaryot Microbiol.

[41] Ikeda-Ohtsubo W, Desai M, Stingl U, Brune A (2007). Phylogenetic diversity of ‘Endomicrobia’ and their specific affiliation with termite gut flagellates.. Microbiology.

[42] Noda S, Kitade O, Inoue T, Kawai M, Kanuka M (2007). Cospeciation in the triplex symbiosis of termite gut protists (*Pseudotrichonympha* spp.), their hosts, and their bacterial endosymbionts.. Mol Ecol.

[43] Carpenter KJ, Chow L, Keeling PJ (2009). Morphology, phylogeny, and diversity of *Trichonympha* (Parabasalia: Hypermastigida) of the wood-feeding cockroach *Cryptocercus punctulatus*.. J Eukaryot Microbiol.

[44] Harper JT, Gile GH, James ER, Carpenter KJ, Keeling PJ (2009). The inadequacy of morphology for species and genus delineation in microbial eukaryotes: an example from the parabasalian termite symbiont *Coronympha*.. PLoS One.

[45] Strassert JFH, Desai MS, Brune A, Radek R (2009). The true diversity of devescovinid flagellates in the termite *Incisitermes marginipennis*.. Protist.

[46] Noda S, Mantini C, Bordereau C, Kitade O, Dolan MF (2009). Molecular phylogeny of parabasalids with emphasis on the order Cristamonadida and its complex morphological evolution.. Mol Phylogenet Evol.

[47] Ohkuma M, Noda S, Hongoh Y, Nalepa CA, Inoue T (2009). Inheritance and diversification of symbiotic trichonymphid flagellates from a common ancestor of termites and the cockroach *Cryptocercus*.. Proc Biol Sci.

[48] Noda S, Hongoh H, Sato T, Ohkuma M (2009). Complex coevolutionary history of symbiotic Bacteroidales bacteria of various protists in the gut of termites.. BMC Evol Biol.

[49] Carpenter KJ, Horak A, Keeling PJ (2010). Phylogenetic position and morphology of Spirotrichosomidae (Parabasalia): new evidence from *Leptospironympha* of *Cryptocercus punctulatus*.. Protist.

[50] Desai MS, Strassert JFH, Meuser K, Hertel H, Ikeda-Ohtsubo W (2010). Strict cospeciation of devescovinid flagellates and Bacteroidales ectosymbionts in the gut of dry-wood termites (Kalotermitidae).. Environ Microbiol.

[51] Viscogliosi E, Müller M (1998). Phylogenetic relationships of the glycolytic enzyme, glyceraldehydes-3-phosphate dehydrogenase, from parabasalid flagellates.. J Mol Evol.

[52] Keeling PJ, Palmer JD (2000). Parabasalian flagellates are ancient eukaryotes.. Nature.

[53] Keeling PJ (2004). Polymorphic insertions and deletions in parabasalian enolase genes.. J Mol Evol.

[54] Gerbod D, Sanders EP, Moriya S, Noël C, Takasu H (2004). Molecular phylogenies of Parabasalia inferred from four protein genes and comparison with rRNA trees.. Mol Phylogenet Evol.

[55] Ohkuma M, Saita K, Inoue T, Kudo T (2007). Comparison of four protein phylogeny of parabasalian symbionts in termite guts.. Mol Phylogenet Evol.

[56] Hauck R, Hafez HM (2010). Systematic position of *Histomonas meleagridis* based on four protein genes.. J Parasitol.

[57] Malik S-B, Brochu CD, Bilic I, Yuan J, Hess M (2011). Phylogeny of parasitic Parabasalia and free-living relatives inferred from conventional markers vs. *Rpb1*, a single-copy gene.. PLoS One.

[58] Brugerolle G (1976). Cytologie ultrastructurale, systématique et évolution des Trichomonadida.. Ann Stat Biol de Besse-en-Chandesse.

[59] Honigberg BM (1963). Evolutionary and systematic relationships in the flagellate order Trichomonadida Kirby.. J Protozool.

[60] Brugerolle G, Patterson DJ (2001). Ultrastructure of Joenina pulchella Grassi, 1917 (Protista, Parabasalia), a reassessment of evolutionary trends in the parabasalids, and a new order Cristamonadida for devescovinid, calonymphid and lophomonad flagellates.. Org Divers Evol.

[61] Adl SM, Simpson AG, Farmer MA, Andersen RA, Anderson OR (2005). The new higher level classification of eukaryotes with emphasis on the taxonomy of protists.. J Eukaryot Microbiol.

[62] Liapounova NA, Hampl V, Gordon PMK, Sensen CW, Gedamu L (2006). Reconstructing the mosaic glycolytic pathway of the anaerobic eukaryote *Monocercomonoides*.. Eukaryot Cell.

[63] Stechmann A, Baumgartner M, Silberman JD, Roger AJ (2006). The glycolytic pathway of *Trimastix pyriformis* is an evolutionary mosaic.. BMC Evol Biol.

[64] Rogers MB, Watkins RF, Harper JT, Durnford DG, Gray MW (2007). A complex and punctate distribution of three eukaryotic genes derived by lateral gene transfer.. BMC Evol Biol.

[65] Delsuc F, Brinkmann H, Philippe H (2005). Phylogenomics and the reconstruction of the tree of life.. Nat Rev Genet.

[66] Hillis DM, Pollock DD, McGuire JA, Zwickl DJ (2003). Is sparse taxon sampling a problem for phylogenetic inference?. Syst Biol.

[67] Rosenberg MS, Kumar S (2003). Taxon sampling, bioinformatics, and phylogenomics.. Syst Biol.

[68] Todaka N, Inoue T, Saita K, Ohkuma M, Nalepa CA (2010). Phylogenetic analysis of cellulolytic enzyme genes from representative lineages of termites and a related cockroach.. PLoS One.

[69] Todaka N, Moriya S, Saita K, Hondo T, Kiuchi I (2007). Environmental cDNA analysis of the genes involved in lignocellulose digestion in the symbiotic protist community of *Reticulitermes speratus*.. FEMS Microbiol Ecol.

[70] Hollande A, Carruette-Valentin J (1971). Les atractophores, l'induction du fuseau et la division cellulaire chez les hypermastigines. Etude infrastructurale et révision systématique des Trichonymphines et des Spirotrichonymphines.. Protistologica.

[71] Kirby H (1947). Flagellate and host relationships of trichomonad flagellates.. J Parasitol.

[72] Honigberg BM, Mattern CF, Daniel WA (1971). Fine structure of the mastigont system in *Tritrichomonas foetus* (Riedmuller).. J Protozool.

[73] Carlton JM, Hirt RP, Silva JC, Delcher AL, Schatz M (2007). Draft genome sequence of the sexually transmitted pathogen *Trichomonas vaginalis*.. Science.

[74] Bricheux G, Brugerolle G (1997). Molecular cloning of actin genes in *Trichomonas vaginalis* and phylogeny inferred from actin sequences.. FEMS Microbiol Lett.

[75] Roger AJ, Sandblom O, Doolittle WF, Philippe H (1999). An evaluation of elongation factor 1 alpha as a phylogenetic marker for eukaryotes.. Mol Biol Evol.

[76] Moriya S, Tanaka K, Ohkuma M, Sugano S, Kudo T (2001). Diversification of the microtubule system in the early stage of eukaryote evolution: elongation factor 1α and α-tubulin protein phylogeny of termite symbiotic oxymonad and hypermastigote protists.. J Mol Evol.

[77] Bilic I, Leberl M, Hess M (2009). Identification and molecular characterization of numerous *Histomonas meleagridis* proteins using a cDNA library.. Parasitology.

[78] Hedtke SM, Townsend TM, Hillis DM (2006). Resolution of phylogenetic conflict in large data sets by increased taxon sampling.. Syst Biol.

[79] Brugerolle G (2001). Morphological characters of spirotrichonymphids: *Microjoenia*, *Spirotrichonymphella* and *Spirotrichonympha* symbionts of the Australian termite *Porotermes grandis*.. Eur J Protistol.

[80] Gile GH, Slamovits CH (2011). Phylogenetic position of *Lophomonas striata* Bütschli (Parabasalia) from the hindgut of the cockroach *Periplaneta americana*.. Protist.

[81] Dolan M, Margulis L (1997). *Staurojoenina* and other symbionts in *Neotermes* from San Salvador Island, Bahamas.. Symbiosis.

[82] Hollande A (1986). Les hypermastigines du genre *Staurojoenina*. Précisions sur la structure de leur rostre et description d'espèces nouvelles.. Protistologica.

[83] Viscogliosi E, Brugerolle G (1994). Striated fibers in trichomonads: costa proteins represent a new class of proteins forming striated roots.. Cell Motil Cytoskeleton.

[84] Cepicka I, Kutišová K, Tachezy J, Kulda J, Flegr J (2005). Cryptic species within the *Tetratrichomonas gallinarum* species complex revealed by molecular polymorphism.. Vet Parasitol.

[85] Diamond LS (1957). The establishment of various trichomonads of animals and man in axenic cultures.. J Parasitol.

[86] Viscogliosi E, Philippe H, Baroin A, Perasso R, Brugerolle G (1993). Phylogeny of trichomonads based on partial sequences of large subunit rRNA and on cladistic analysis of morphological data.. J Eukaryot Microbiol.

[87] Kitade O, Maeyama T, Matsumoto T (1997). Establishment of symbiotic flagellate fauna of *Hodotermopsis japonica* (Isoptera: Termopsidae).. Sociobiology.

[88] Noda S, Iida T, Kitade O, Nakajima H, Kudo T (2005). Endosymbiotic *Bacteroidales* bacteria of the flagellated protist *Pseudotrichonympha grassii* in the gut of the termite *Coptotermes formosanus*.. Appl Environ Microbiol.

[89] Noda S, Kawai M, Nakajima H, Kudo T, Ohkuma M (2006). Identification and *in situ* detection of two lineages of *Bacteroidales* ectosymbionts associated with a termite gut protist, *Oxymonas* sp.. Microbes Environ.

[90] Hongoh Y, Sato T, Dolan MF, Noda S, Ui S (2007). The motility symbiont of the termite gut flagellate *Caduceia versatilis* is a member of the “*Synergistes*” group.. Appl Environ Microbiol.

[91] Inoue J-I, Noda S, Hongoh Y, Ui S, Ohkuma M (2008). Identification of endosymbiotic methanogen and ectosymbiotic spirochetes of gut protists of the termite *Coptotermes formosanus*.. Microbes Environ.

[92] Larkin MA, Blackshields G, Brown NP, Chenna R, McGettigan PA (2007). Clustal W and Clustal X version 2.0.. Bioinformatics.

[93] Abascal F, Zardoya R, Posada D (2005). ProtTest: selection of best-fit models of protein evolution.. Bioinformatics.

[94] Stamatakis A (2006). RAxML-VI-HPC: maximum likelihood-based phylogenetic analyses with thousands of taxa and mixed models.. Bioinformatics.

[95] Huelsenbeck JP, Ronquist F (2001). MRBAYES: Bayesian inference of phylogenetic trees.. Bioinformatics.

[96] Schmidt HA, Strimmer K, Vingron M, von Haeseler A (2002). TREE-PUZZLE: maximum likelihood phylogenetic analysis using quartets and parallel computing.. Bioinformatics.

[97] Lartillot N, Brinkmann H, Philippe H (2007). Suppression of long-branch attraction artifacts in the animal phylogeny using a site-heterogeneous model.. BMC Evol Biol.

[98] Shimodaira H, Hasegawa M (2001). CONSEL: for assessing the confidence of phylogenetic tree selection.. Bioinformatics.

