# Supplementary figures and images for: Molecular Phylogeny and Evolution of Parabasalia with Improved Taxon Sampling and New Protein Markers of Actin and Elongation Factor-1α
Source: PLoS One. 2012 Jan 9;7(1):e29938. doi: 10.1371/journal.pone.0029938 (PMC3253790; doi:10.1371/journal.pone.0029938)

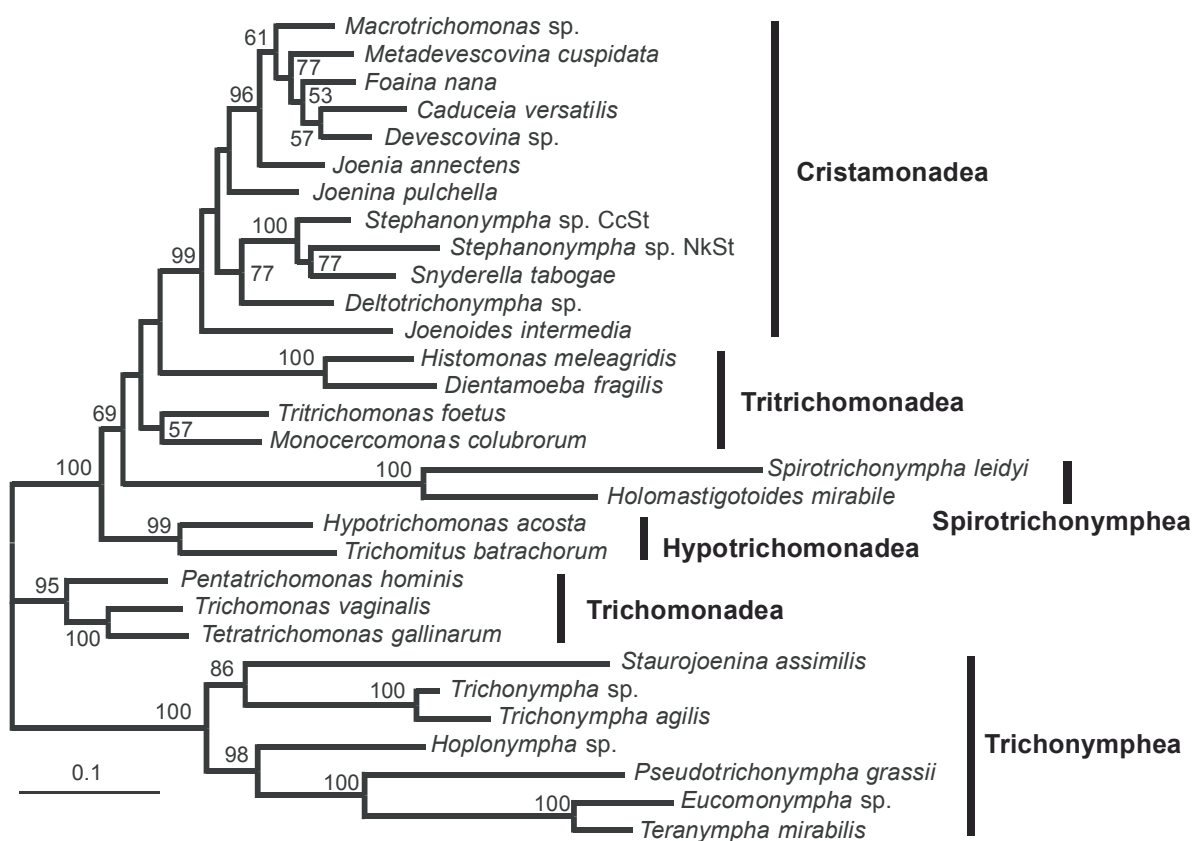

Supplement: Figure S1 — Exclusion of GAPDH from the sequence concatenation of parabasalids. The GAPDH sequence was excluded from the dataset used for the inference shown in Figure 2, and the sequences of Deltotrichonympha sp. and Dientamoeba were added to the analysis. The tree was inferred using RAxML with the parameters and branch length optimized for each partition. The bootstrap values above 50% are indicated at the nodes. Note that the relationship of the six parabasalian classes and the root position did not change after the exclusion except for the paraphyly of Tritrichomonadea. (PDF) [file pone.0029938.s001.pdf]

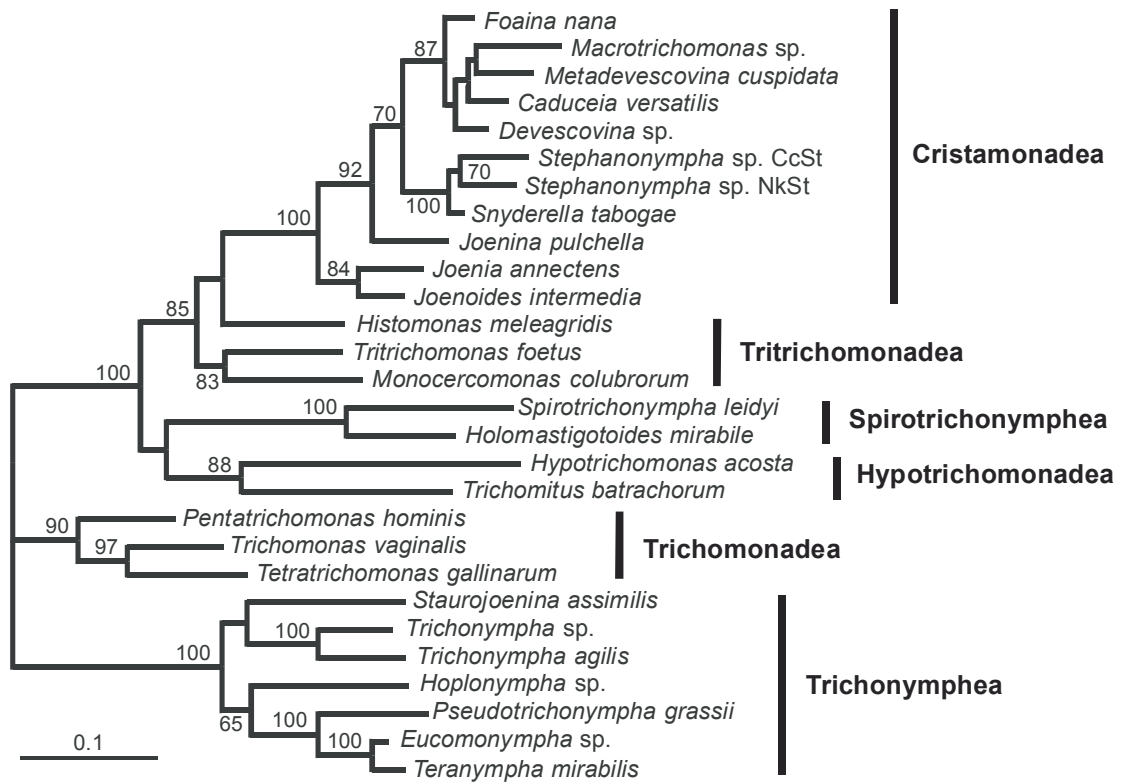

Supplement: Figure S2 — Exclusion of SSU rRNA gene from the sequence concatenation of parabasalids. The SSU rRNA gene sequence was excluded from the dataset used for the inference shown in Figure 2, and the tree was inferred using RAxML with the parameters and branch length optimized for each partition. The bootstrap values above 50% are indicated at the nodes. Note that the relationship of the six parabasalian classes and the root position did not change after the exclusion except for the paraphyly of Tritrichomonadea and the sister-group relationship of Spirotrichonymphea and Hypotrichomonadea. The removal of the SSU rRNA gene sequence was particularly important because the base composition of this gene was heterogeneous in the two Spirotrichonymphea members and Histomonas. All taxa were homogeneous in amino acid composition in the three protein sequences. (PDF) [file pone.0029938.s002.pdf]

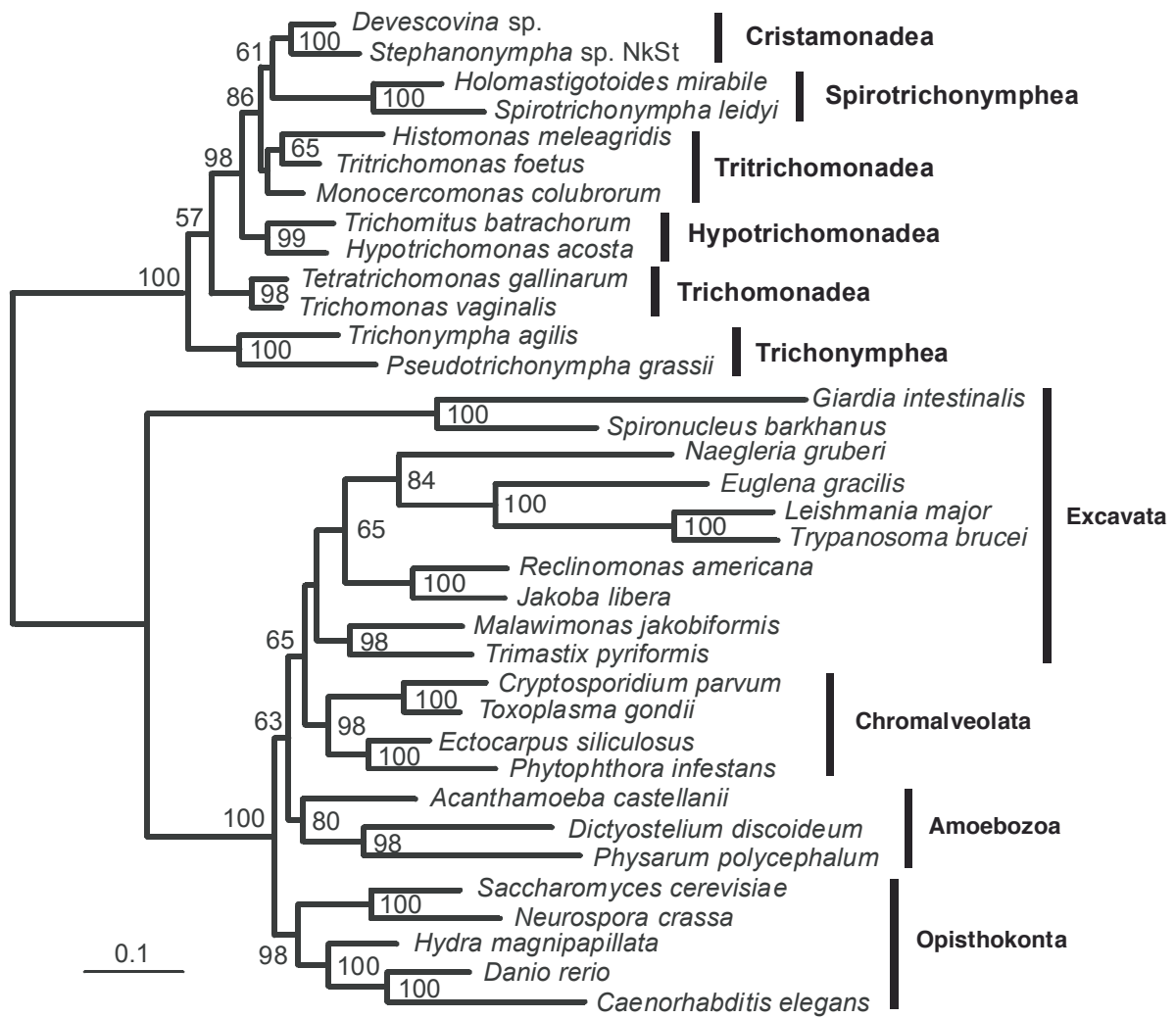

Supplement: Figure S3 — Maximum likelihood tree of the sequence concatenation of actin, EF-1α, α-tubulin, β-tubulin, and SSU rRNA gene in 13 parabasalian taxa and outgroup eukaryotes. Unambiguously aligned sites of α-tubulin (351) and β-tubulin (145) were concatenated with the dataset used for the analysis shown in Figure 3. The sequence data for both tubulins used for the concatenation are the same as described previously [55]. The tree was inferred using RAxML with the parameters and branch length optimized for each partition. The bootstrap values above 50% are indicated at the nodes. Because the sequences of α- and β-tubulin available for Pentatrichomonas and Entamoeba are short, we excluded them from the analysis. Trichonymphea was the most basal parabasalian lineage in this analysis; however, this rooting was likely a wrong inference caused by the limited taxon sampling (see the main text). Spirotrichonymphea instead of Tritrichomonadea was sister to Cristamonadea, but this change was not supported at all. The taxa exclusion analyses (see Table 4) indicated that when the number of parabasalian taxa was reduced, the frequency of the pairing of Cristamonadea and Spirotrichonymphea increased up to four times in 10 repeated analyses, suggesting an artificial pairing owing to the limited number of examined taxa. (PDF) [file pone.0029938.s003.pdf]
