# Supplementary material for: Molecular Phylogeny and Evolution of Parabasalia with Improved Taxon Sampling and New Protein Markers of Actin and Elongation Factor-1α
Source: PLoS One. 2012 Jan 9;7(1):e29938. doi: 10.1371/journal.pone.0029938 (PMC3253790; doi:10.1371/journal.pone.0029938)
Supplement: Table S1 — Removal of long-branch outgroup taxa and the effect on bootstrap values for major nodes within Parabasalia. (DOC) [file pone.0029938.s004.doc]

Table S1. Removal of long-branch outgroup taxa and the effect on bootstrap values for major nodes within Parabasalia.

| Node^a^ | Number of outgroup taxa removed^b^ | | | | | | | | | | |
| --- | --- | --- | --- | --- | --- | --- | --- | --- | --- | --- | --- |
|  | 0 | 1 | 2 | 3 | 4 | 5 | 6 | 7 | 8 | 9 | 10 |
| a | 99 | 99 | 99 | 99 | 99 | 99 | 99 | 100 | 99 | 99 | 99 |
| b | 100 | 100 | 100 | 100 | 100 | 100 | 100 | 100 | 100 | 100 | 100 |
| c | 35 | 34 | 40 | 39 | 38 | 33 | 34 | 37 | 0 | 30 | 36 |
| d | 100 | 100 | 100 | 100 | 100 | 100 | 100 | 100 | 100 | 100 | 100 |
| e | 97 | 96 | 96 | 97 | 97 | 95 | 96 | 94 | 92 | 94 | 96 |
| f | 93 | 90 | 90 | 89 | 88 | 92 | 91 | 95 | 93 | 96 | 94 |
| g | 100 | 100 | 100 | 100 | 100 | 100 | 100 | 100 | 100 | 100 | 100 |
| h | 25 | 29 | 33 | 33 | 32 | 28 | 29 | 31 | 0 | 22 | 28 |
| i | 42 | 42 | 45 | 48 | 44 | 43 | 45 | 47 | 0 | 40 | 44 |
| j | 53 | 46 | 56 | 57 | 61 | 54 | 58 | 60 | 55 | 54 | 55 |
| k | 90 | 92 | 90 | 93 | 94 | 92 | 89 | 84 | 85 | 81 | 86 |
| l | 74 | 68 | 60 | 61 | 72 | 80 | 79 | 82 | 77 | 84 | 80 |
| m | 100 | 100 | 100 | 100 | 100 | 100 | 100 | 100 | 100 | 100 | 100 |

The distance from the inferred root position to each outgroup taxon was calculated in the ML tree shown in Figure 3 using TreeStat (http://tree.bio.ed.ac.uk/software/treestat/). The longest branched outgroup taxon was progressively excluded from the bootstrap analyses of 100 replicates using RAxML with the CAT model (GTRCAT for SSU rRNA gene and PROTCATWAG for the protein sequences). Note that the root position did not change through the removal analyses and that the bootstrap support values for this rooting were substantially stable (see nodes k and l).

^a^ Nodes a to j are those indicated in Figure 3. Node k corresponds to the grouping of Cristamonadea, Tritrichomonadea, Spirotrichonymphea, and Hypotrichomonadea, whereas node l to the grouping of Trichomonadea and Trichonymphea. Node m corresponds to the monophyly of Parabasalia.

^b^ Outgroup taxa removed progressively were 1, *Giardia*; 2, *Trypanosoma*; 3, *Leishmania*; 4, *Entamoeba*; 5, *Spironucleus*; 6, *Euglena*; 7, *Physarum*; 8, *Naegleria*; 9, *Caenorhabditis*; and 10, *Trimastix*.
