# Supplementary material for: Molecular Phylogeny and Evolution of Parabasalia with Improved Taxon Sampling and New Protein Markers of Actin and Elongation Factor-1α
Source: PLoS One. 2012 Jan 9;7(1):e29938. doi: 10.1371/journal.pone.0029938 (PMC3253790; doi:10.1371/journal.pone.0029938)
Supplement: Table S2 — Species used for the outgroup and their sequence accession numbers used for the concatenation. (DOC) [file pone.0029938.s005.doc]

**Table S2. Species used for the outgroup and their sequence accession numbers used for the concatenation.**

| Species | SSU rRNA | Actin | | EF-1α | | α-tubulin | | β-tubulin | |
| --- | --- | --- | --- | --- | --- | --- | --- | --- | --- |
| *Giardia lamblia* | NW_001844081 | EDO76979 | | XP_001704547 | | XP_001706843 | | XP_001707388 | |
| *Spironucleus barkhanus* | AY646679 | | EST data^a^ | | AAC47211 | | AAC47209 | | EST data^a^ |
| *Trimastix pyriformis* | AF244903 | | TPL00000260 | | ABC54653 | | TPL00000212 | | TPL00001398 |
| *Malawimonas jakobiformis* | EF455761 | | ABX25969 | | ABC54649 | | AF267181 | | AF267185 |
| *Naegleria gruberi* | M18732 | | AAF37002 | | ABC54650 | | CAA56939 | | XP_002669519 |
| *Euglena gracilis* | M12677 | | AAC99646 | | ACO50110 | | AAK37832 | | AAK37834 |
| *Leishmania major* | NC_007268 | | AAA19789 | | XP_001682264 | | CAJ02503 | | CAJ06135 |
| *Trypanosoma brucei* | M12676 | | XP_828467 | | XP_828111 | | XP_001218934 | | XP_001218933 |
| *Reclinomonas americana* | AY117417 | | AAX09575 | | ABC54651 | | AF267182 | | AF267190 |
| *Jakoba libera* | AY117418 | | JLL00000765 | | ACO50113 | | JLL00000756 | | JLL00000772 |
| *Cryptosporidium parvum* | AF164102 | | AAM28417 | | XP_00138834 | | XP_625871 | | XP_627803 |
| *Toxoplasma gondii* | U03070 | | CAJ20602 | | CAJ20335 | | XP_002364807 | | AAA30146 |
| *Phytophthora infestans* | NW_003303738 | | XP_002898250 | | XP_002905383 | | EEY54372 | | XP_002908783 |
| *Ectocarpus siliculosus* | L43062 | | CBJ30601 | | CBJ32894 | | CBJ28184 | | CBN79445 |
| *Entamoeba histolytica* | X64142 | | AAA29085 | | XP_651869 | | – | | – |
| *Dictyostelium discoideum* | AM168071 | | XP_636169 | | XP_645978 | | XP_637058 | | XP_646162 |
| *Physarum polycephalum* | X13160 | | CAA30629 | | AAB69706 | | CAA28712 | | AAA29974 |
| *Acanthamoeba castellanii* | AF260724 | | CAA23399 | | AAU94656 | | AAZ80770 | | AAZ80771 |
| *Saccharomyces cerevisiae* | HQ174900 | | AAA34391 | | CAA55620 | | AAA35180 | | CAA24603 |
| *Neurospora crassa* | X04971 | | AAC78496 | | XP_964868 | | EAA29668 | | EAA28433 |
| *Hydra magnipapillata* | HQ392522 | | XP_002154696 | | XP_002160595 | | XP_002159229 | | XP_00216191 |
| *Danio rerio* | BX537263 | | AAO38846 | | NP_571338 | | NP_919369 | | NP_942104 |
| *Caenorhabditis elegans* | EU196001 | | CAA34720 | | P53013 | | BAA03909 | | NP_499367 |

^a^ The EST sequences in the public database (GW585169-GW589878, and [EC585128](http://www.ncbi.nih.gov/entrez/query.fcgi?db=Nucleotide&cmd=search&term=EC585128)-[EC586011](http://www.ncbi.nih.gov/entrez/query.fcgi?db=Nucleotide&cmd=search&term=EC586011)) were searched and assembled to produce in silico-translated amino acid sequence.
